# Mixed method program impact evaluation: Reducing economic barriers to accessing health services (REBAHS) long-term primary healthcare subsidization protocol (LPSP) II action in Lebanon

**DOI:** 10.1371/journal.pgph.0005569

**Published:** 2025-12-05

**Authors:** Fadi El-Jardali, Jade Khalife, Clara Abou Samra, Sahar Nassour, Natalya Kostandova, Brett Collins

**Affiliations:** 1 Department of Health Management and Policy, Faculty of Health Sciences, American University of Beirut, Beirut, Lebanon; 2 Higher Institute of Public Health, Saint-Joseph University of Beirut, Beirut, Lebanon; 3 Knowledge to Policy Center, Faculty of Health Sciences, American University of Beirut, Beirut, Lebanon; 4 International Medical Corps, Washington, DC, United States of America; Qatar University College of Medicine, QATAR

## Abstract

The REBAHS LPSP II action launched in May 2022 by International Medical Corps (IMC) and Première Urgence Internationale (PUI) in Lebanon is a continuation of previous initiatives implemented in Primary Healthcare Centers (PHCCs) with the key aim to support service provision in PHCCs. This study aims to present a multi-faceted impact evaluation approach, quantifying the outcomes of the action and identifying areas for improvement. A mixed-method approach was adopted, including quantitative and qualitative tools to assess the impact of the initiative’s health sector. Indicators of PHCC service utilization were extracted from the National Health Information System (PHENICS) and analyzed through descriptive analysis, interrupted time series analysis, and cost-effectiveness analysis. Key-informant interviews (KII) were conducted with donors and stakeholders, and Focus Group Discussions (FGDs) were implemented with PHCC staff members to gain a deeper understanding of the impact, challenges, and areas for improvement of the initiative. The action resulted in an overall increase in the proportion of children being screened for malnutrition, women being screened for breast cancer via mammogram testing, and a high number of antenatal care visits. Improved access to care, patient satisfaction, quality of care, and disease detection and management among other positive impacts have been highlighted. Barriers to implementation including administrative burdens, staff turnover, and financial constraints among others have been suggested. Lessons learned and recommendations for future program design have been highlighted. This study may serve as a model for enhancing healthcare systems globally by enhancing global funding models, optimizing resource allocation, and improving program impact, specifically in conflict-affected settings and countries with limited resources, thereby enhancing healthcare access, health service delivery, and program sustainability.

## Introduction

### Background

Recent years have demonstrated a renewed global commitment to universal health coverage (UHC) [1]. The World Health Organization (WHO) constitution in 1946 declared the enjoyment of the highest possible standard of health as a fundamental human right [2]. The Alma-Ata declaration set in 1978 has further emphasized this right through the Health for All Agenda [3]. The UN has designated universal UHC as one of the health-related Sustainable Development Goals (SDGs) to be accomplished by 2030, declaring that every individual should have the right to receive the needed healthcare services without financial hardship [4]. The Astana Declaration has furthered the commitment to healthcare as a human right, inextricably linking UHC to primary health care (PHC) [5].

Estimates suggest that the global burden of disease can be reduced by 70% through PHC services potentially meeting the needs of 80% of the population [1]. PHCs have been given further attention due to their exceptional capacity to target the most vulnerable populations, which is crucial in ensuring health equity across the nations. A sustainable essential benefits package (EBP) for PHCs is a stepping stone towards ensuring population health and achieving UHC [1]. This includes a set of defined essential health services that are accessible to all the population. A limited benefit package can be delivered before prioritization and subsequent expansion of services upon resource availability [4].

### REBAHS LPSP II action in Lebanon

The Reducing Economic Barriers to Accessing Health Services (REBAHS) project was initiated in 2018 through a consortium of international non-governmental organizations (INGOs) to reduce the burden of healthcare costs on people impacted by the Syrian Refugee Crisis in Lebanon. REBAHS supports specific primary healthcare centers (PHCCs) through subsidized services based on a flat fee model that requires patients to pay US$2 for consultations while supporting INGOs to cover the remaining visit costs [6]. The initiative was implemented over 26 months from January 2018 to February 2020 [7].

The Beirut Port Explosion in August 2020 magnified the already pressing need to enhance access to PHC services by directly subsidizing services to reduce or eliminate out-of-pocket expenses in Lebanon. As part of the response efforts, the “National Primary Health Care Network Appeal for the Beirut Port Explosion Response” and the “Immediate Response Model” (IRM) were developed as interim joint standardized protocols to cover primary healthcare services in Lebanon. A Task Force was established to define the Essential Benefit Package (EBP) which provides services delivered as part of a long-term primary healthcare subsidization protocol (LPSP), based on the REBAHS model [6].

**Fig 1:**
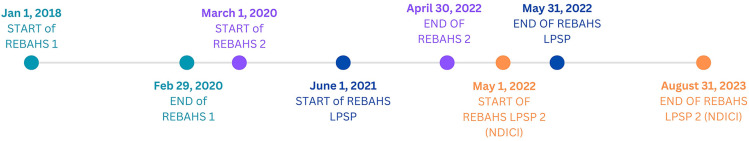
REBAHS and LPSP timeline.

In response to the pressing need for healthcare services in Lebanon, the REBAHS LPSP II action was launched in May 2022 by the International Medical Corps (IMC) and Première Urgence Internationale (PUI) as a continuation of the REBAHS initiative (Fig 1). This action aimed to provide immediate assistance to vulnerable populations, support service provision through national systems, reinforce Lebanon’s stability, and target Syrian refugees and other vulnerable groups. It also focused on strengthening local community capacity and building on previous initiatives to integrate mental health into primary healthcare.

IMC and PUI subsidized services offered at PHCCs as part of a package model under the REBAHS LPSP II initiative. Services included but were not limited to consultations, follow-ups, vaccination, counseling, and medical tests under the LPSP Wellness Packages, Safe Motherhood LPSP packages (e.g., Antenatal care and postpartum family planning), Non-Communicable Diseases (NCD) Applicable LPSP Packages, Common Conditions Packages, and support for People with Disabilities (PwD) (e.g., diagnostic tests and rehabilitation services) (S1 Appendix).

Under the services agreement between IMC, PUI, and the PHCCs, critical aspects were considered including consultation fees, payment terms, prices, and discounted rates to ensure unhindered access to essential health services for Persons of Concern (POC). Oversight and supervision involved regular check-ups and monitoring by IMC/PUI health staff available at the PHCC to ensure quality care. Accordingly, IMC, PUI, and the Clinic developed improvement plans aligned with the Ministry of Public Health (MoPH) and WHO standards to improve service delivery. IMC and PUI provided mentoring and technical support to the clinic to ensure the effective implementation of the developed plan.

This study aims to present a multi-faceted impact evaluation approach, quantifying the outcomes of the REBAHS LPSP II action, identifying areas for improvement, measuring the success of efforts, evaluating scalability potential, and demonstrating accountability to stakeholders. It will further serve to provide valuable insights and high-level recommendations for future programming, enabling informed decision-making and planning, fostering transparency and sustainability, mitigating potential adverse consequences, optimizing resource allocation, and amplifying the overall impact of IMC’s and PUI’s work.

## Materials and methods

### Ethics statement

The program evaluation protocol (e.g., objectives, recruitment, data collection, and analysis methods) was approved by the Institutional Review Board (IRB) (Protocol Number: SBS-2023-0285) at the American University of Beirut before engaging with the participants. Participants were invited via E-mail whereby a consent script approved by the IRB was shared. Participants were requested to read the consent form and approve it by E-mail prior to participating in the study. Each FGD and KII were conducted by two research team members. At the beginning of each FGD and KII the consent form was read outloud to participants and oral consent was sought for participation and audio recording. Verbal consent was witness by the second interviewer. Participants were informed that the findings from the discussions will be presented anonymously. Data sharing adhered to strict protocols, whereby only aggregate data without personally identifiable information (PII) was provided by authorized personnel to the research team for data analysis.

### Design

This multifaceted research used a mixed-method approach including both quantitative and qualitative tools to assess the impact of REBAHS LPSP II’s health sector initiative. Extensive documentation review of IMC’s reports, assessment tools, package coverage, satisfaction surveys, and databases have been conducted in the first phase. IMC facilitated the communication between the research team and the MoPH focal points, who extracted relevant data and indicators from the Health Information System (PHENICS) used on behalf of the PHCCs and at the national level. The extracted data was shared with IMC for approval and then shared with the research team for analysis on June 2023. Extracted data encompassed aggregated data without personally identifiable information (PII) to ensure patients privacy.

The second phase included a series of assessment techniques including descriptive analysis, interrupted time series (ITS) analysis of key indicators, cost-effectiveness analysis of essential services provided at the PHCCs, key-informant interviews (KII) with donors and stakeholders, and Focus Group Discussions (FGDs) with PHCC staff members. The analysis has been implemented in line with the systems thinking framework of primary healthcare governance and delivery at the individual, primary care center, and health system levels [8]. The mixed-methods approach enables a deeper understanding and validation of the findings through the integration of qualitative and quantitative data.

#### Individual and primary healthcare-level analysis.

Consultations were conducted with the team from IMC to develop an overall understanding of the REBAHS LPSP II initiative. Consequently, a list of key performance indicators was shared by IMC. Several rounds of review were conducted to finalize the list of indicators and ensure the scope and intention of measurement fit the context of this evaluation. A total of 21 outcome, process, and structure key performance indicators were identified in line with the Donabedian theory after close consideration of the data available in PHENICS [9]. Indicators 1–18 were deemded as process indicators, while those from 19 to 21 were deemed as structure indicators. Denominators were identified according to the volume of consultations conducted at PHCCs as catchment population estimates were not available (Table 1).

**Table 1:** Individual and PHCC level indicators.

#	Indicator theme	Indicator Level	Numerator	Denominator
**1**	Hypertension (first visit)	PHCC Level	Adult persons (≥18 years) enrolled in the hypertension package, having their first visit to the PHCC, per calendar month.	Adult persons (≥18 years) visiting PHCCs for the first time, for any service, per calendar month.
**2**	Hypertension (second visit)	PHCC Level	Adult persons (≥18 years) enrolled in the hypertension package, having their second visit to the PHCC, per calendar month.	Adult persons (≥18 years) visiting PHCCs for the second time, for any service, per calendar month.
**3**	Diabetes (first visit)	PHCC Level	Adult persons (≥18 years) enrolled in the diabetes package, having their first visit to the PHCC, per calendar month.	Adult persons (≥18 years) visiting PHCCs for the first time, for any service, per calendar month.
**4**	Diabetes (second visit)	PHCC Level	Adult persons (≥18 years) enrolled in the diabetes package, having their second visit to the PHCC, per calendar month.	Adult persons (≥18 years) visiting PHCCs for the second time, for any service, per calendar month.
**5**	Coronary artery disease (first visit)	PHCC Level	Adult persons (≥18 years) enrolled in the CAD package, having their first visit to the PHCC, per calendar month.	Adult persons (≥18 years) visiting PHCCs for the first time, for any service, per calendar month.
**6**	Coronary artery disease (second visit)	PHCC Level	Adult persons (≥18 years) enrolled in the CAD package, having their second visit to the PHCC, per calendar month.	Adult persons (≥18 years) visiting PHCCs for the second time, for any service, per calendar month.
**7**	Chronic obstructive pulmonary disease (first visit)	PHCC Level	Adult persons (≥18 years) enrolled in the COPD package, having their first visit to the PHCC, per calendar month.	Adult persons (≥18 years) visiting PHCCs for the first time, for any service, per calendar month.
**8**	Chronic obstructive pulmonary disease (second visit)	PHCC Level	Adult persons (≥18 years) enrolled in the COPD package, having their second visit to the PHCC, per calendar month.	Adult persons (≥18 years) visiting PHCCs for the second time, for any service, per calendar month.
**9**	Lipid profile	Individual Level	Adult persons aged 40 years or older, having a full fasting lipid profile at PHCC, per calendar month.	Adult persons aged 40 years or older, visiting PHCCs for the first time, for any service, per calendar month.
**10**	Mammogram screening for breast cancer	Individual Level	Women aged 40 years or older, having a mammogram to screen for breast cancer, per calendar month.	Women aged 40 years or older visiting PHCCs for the first time, for any service, per calendar month.
**11**	Pap smear screening for cervical cancer	Individual Level	Women aged between 21 and 64 years, having a Pap smear to screen for cervical cancer, per calendar month.	Women aged between 21 and 64 years, visiting PHCCs for the first time, for any service, per calendar month.
**12**	Measles, Mumps, and Rubella (MMR) vaccination	Individual Level	Children between the ages of 0- and 5-years receiving MMR vaccination at PHCC, per calendar month.	Children between the ages of 0 and 5 years visiting PHCCs for the first time, for any service, per calendar month.
**13**	Malnutrition Screening	Individual Level	Children between the ages of 0 and 5 years screened for malnutrition at PHCC, per calendar month.	Children between the ages of 0 and 5 years visiting PHCCs for the first time, for any service, per calendar month.
**14**	Antenatal care counseling (first consultation)	Individual Level	Girls and women aged 16 years or older, having their first ANC visit at PHCC, during their first trimester, per calendar month.	Girls and women aged 16 years or older, visiting PHCC for the first time, for any service during their first trimester of pregnancy, per calendar month.
**15**	Antenatal care counseling (fourth consultation)	Individual Level	Girls and women aged 16 years or older, having their fourth ANC visit at PHCC, during their first trimester, per calendar month.	Girls and women aged 16 years or older, visiting PHCC for the first time, for any service during their first trimester of pregnancy, per calendar month.
**16**	Antenatal care counseling (eighth consultation)	Individual Level	Girls and women aged 16 years or older, having their eighth ANC visit at PHCC, during their first trimester, per calendar month.	Girls and women aged 16 years or older, visiting PHCC for the first time, for any service during their first trimester of pregnancy, per calendar month.
**17**	Breastfeeding counseling	Individual Level	Girls and women aged 16 years or older, having a breastfeeding counseling visit for the first time at PHCC, per calendar month.	Girls and women aged 16 years or older visiting PHCCs for the first time, for any service, per calendar month.
**18**	Family Planning Counseling	Individual Level	Girls and women aged 16 years or older, having a family planning counseling visit for the first time at PHCC, per calendar month.	Girls and women aged 16 years or older visiting PHCCs for the first time, for any service, per calendar month.
**19**	Nationality	PHCC Level	Persons of any age, visiting PHCC for the first time, stratified by nationality (Lebanese, Syrian, Other), per calendar month.	All persons of any age, visiting PHCC for the first time, per calendar month.
**20**	Sex	PHCC Level	Persons of any age, visiting PHCC for the first time, stratified by sex (Female, Male), per calendar month.	All persons of any age, visiting PHCC for the first time, per calendar month.
**21**	Age	PHCC Level	Persons visiting PHCC for the first time, stratified by age groups (0–2 years, 3–9 years, 10–17 years, 18–44 years, 45–64 years, and more than 64 years), per calendar month.	All persons of any age, visiting PHCC for the first time, per calendar month.

### Quantitative data analysis

#### Descriptive and Interrupted Times Series (ITS) analysis.

Stata software (v.16.1) was utilized to develop specific algorithms to calculate each indicator [10]. At the aggregate level of all individuals seeking care, the indicators were analyzed using descriptive analysis of the time series, and ITS analysis where relevant. The dataset included services delivered between January 2018 and August 2023.

General descriptive analysis was used to assess the indicators. The first figure of each condition included the absolute values of the specific service, the absolute values of a denominator that is useful to contextualize the absolute values of the specific service, and the proportion of the service to the denominator used. The denominator was used as a contextualizing factor, as the exact figure of eligible persons in the population was not available. For most indicators, the term ‘persons’ represents unique individuals (non-repetitive) visiting PHCCs. This figure differs from the term ‘visits’ which can be repetitive. The indicator description table details the criteria used to define specific service and denominator variables.

The changes in indicator levels (abrupt decreases or increases) and trends (gradual increases or decreases) were analyzed using ITS approach (S2 Appendix). Assumptions were identified for this analysis to guard against threats to study validity. Interruption time points were identified after extensive revision of documents provided by IMC and consultations with the IMC team. These include the start of REBAHS LPSP II and the start of previous initiatives conducted by IMC and PUI including REBAHS II and REBAHS LPSP (Fig 1). An evaluation of the start of REBAHS I was not possible due to the limited data in PHENICS. A total of 10 and 11 indicators were identified at the individual and PHCC levels respectively (Table 1).

#### Cost-effectiveness analysis.

Cost-effectiveness analysis was used to evaluate the subsidized services at the PHCC level, by comparing the costs invested to the expected eventual health outcome. The linkages between services provided (i.e., target factor) and associated health outcome were as follows: 1. Hypertension services to decrease burden from hypertensive heart disease, ischemic heart disease and stroke; 2. Diabetes services to decrease burden from complications of diabetes; 3. Mammogram services to decrease burden from breast cancer; 4. Pap smear to decrease burden from cervical cancer; and 5. ANC services to decrease burden from neonatal complications. Cost data was collected from IMC and broken down by individual service packages or services when available. The absolute number of services delivered to generate the program costs by package or service delivered was calculated using data from the PHENICS dataset, and these were mapped downstream to health outcomes using Disability-Adjusted Life Years (DALYs) [11]. Several data sources were used to assess the cost-effectiveness of the package including (1) Estimation of the total cost of the package provided for hypertension, diabetes, CAD, COPD, and ANC, per the reference figures of May 2022 to February 2023; (2) Number of unique persons utilizing primary care centers under the program coverage as calculated through the algorithms using 2018 – 2023 datasets in PHENICS; (3) DALYs for Lebanon, by disease category; (4) Gross Domestic Product (GDP) per capita for Lebanon; (5) Mid-range estimates for the reduction of specific diseases due to package or service delivery; and (6) Mid-range estimates for the population-attributable fraction of certain diseases due to specific risk factors or conditions [11,12].

The total DALYs averted due to the REBAHS LPSP II program were calculated using the WHO’S estimate for DALYs in Lebanon, broken down by disease category, sex, and age; for the program’s timeframe and extended for a ten-year horizon or longer. This included diabetes (diabetes package); hypertensive heart disease, stroke, and ischemic heart disease (hypertension and CAD packages), breast cancer (breast cancer screening), cervical cancer (Pap smear screening), and neonatal conditions (ANC package). The matching population for each of these was also extracted, to calculate the DALY per person, disease, and age. The number of unique persons receiving PHCC services was then used to calculate the DALYs estimated to be averted by the program.

Using the cost breakdown per package or service, the cost per DALY averted for each disease was calculated. These were subsequently totaled to provide the overall cost per DALY averted by the program, as opposed to no other intervention. No discounting was used. The figure was compared to the GDP per capita for Lebanon, using the following general convention:

1. If the cost per DALY averted is less than the GDP per capita, the program is considered to be highly cost-effective.
2. If the cost per DALY averted is between one and three times the GDP per capita, the program is considered to be cost-effective.
3. If the cost per DALY averted is more than three times the GDP per capita, the program is considered to be less cost-effective.

As sensitivity analyses, we varied certain estimates such as disease reduction proportion attributed to the program, with two additional scenarios of assuming program effectiveness was decreased to half, and decreased to a third, relative to the program effectiveness used in the main scenario. A CEA approach was chosen over an impact analysis, as the latter was not possible due to several factors including: 1. Lag time between intervention and disease progression extending beyond program timeline; 2. Contextual factors outside the scope of primary care services (e.g., socioeconomic, environmental); and 3. Information infrastructure limitations which do not allow linkage between primary care and other services received (e.g., hospitalization).

### Qualitative data analysis

#### Study sample.

Study participants were recruited through purposive sampling. A selection criteria was developed to identify a sample of PHCCs across the 5 regions in Lebanon, and other relevant stakeholders (Table 2). Eligibility criteria for the PHCC included geographical location, governance (support provided by IMC or PUI), accreditation status, and the presence of a Primary Healthcare Manager or Director, a physician, and a nurse involved in the REBAHS LPSP II initiative. Purposive selection was done to ensure that the experience of participants captured a wide range of experiences of providers at PHCCs (Table 2).

**Table 2:** Inclusion criteria of study participants.

Stakeholder group	Inclusion criteria
**PHCC Manager and Staff**	• Manager or director contributing to administrative and managerial work, general physician, nurse, and mental health provider (when available) working at a PHCC: • Implementing the REBAHS LPSP II program • Supported by IMC or PUI. • Located in one of the 5 regions of Lebanon (e.g., Beqaa, Beirut, North Lebanon, South Lebanon, Mount Lebanon). • Accredited or not accredited by the MOPH. • Includes Mental Health and Psychosocial Support (MHPSS) or not.
**Decision Maker**	Head of the Primary Health Care Department at the MOPH
**Members of nongovernmental organizations (NGOs) and partner organizations governing PHCs**	Representatives from IMC and PUI involved in the REBAHS LPSP II project.
**Donor agency**	Representatives from donor agencies funding the REBAHS LPSP II project.

#### Data collection.

Participants who took part of the FGDs and the KII were recruited between November 8, 2023 and January 30, 2024. 10 FGDs were conducted with PHCC members each with three to five participants per group including managers, nurses, and general practitioners. FGDs were held virtually via Zoom application in English and/or Arabic. They ranged from 45 to 60 minutes each and were recorded and transcribed verbatim. Discussions followed a semi-structured interview guide (S3 Appendix). At the individual level, questions explored the impact of REBAHS LPSP II on access and quality of care in the PHCCs, the perceived impact on the community across the different benefit packages, successful outcomes achieved through the implementation of the initiative, methods employed to effectively communicate the importance of the benefit packages to the community, quality improvement plans and capacity building activities implemented, key lessons learned, and potential for sustainability. At the health system level, questions explored strategies and practices employed by the PHCCs to manage and monitor medication supply and the supply of medical equipment, effectiveness of capacity-building activities implemented for the staff working on the initiative, challenges faced in implementing the initiative, supply chain management improvement, and changes in the referral system (e.g., protocols and systems in place, challenges).

Four in-depth semi-structured KIIs were conducted with focal points from IMC (n = 1), PUI (n = 1), MoPH (n = 1), and the EU (n = 1). Interviews were held virtually via Zoom application in English and/or Arabic. They ranged from 45 to 60 minutes each and were recorded and transcribed verbatim. Discussions followed two predetermined interview guides designed to assess the impact of REBAHS LPSP II at the system level (S4 Appendix). Questions targeted at representatives from IMC, PUI and the EU were designed to explore the engagement of PHCCs in program design activities, alignment of primary program activities (e.g., fund management, program, design, and delivery) with the objectives of the PHCCs and communities they serve, challenges and barriers faced by the PHCCs in optimizing the value chain, prioritization and allocation of funds to different initiatives, data collection and monitoring systems in place, and collaborations and coordination’s with local NGOs to support the delivery of the program. Additional questions addressed strategies for sustainability, the support provided to PHCCs to overcome program implementation challenges, capacity building support, the key success of the program, implementation challenges, key lessons learned, and the potential for program strengthening. Questions targeted to the head of the MoPH explored collaborations with other relevant stakeholders to strengthen the value chain (e.g., human resource management, financing, supply chain management), challenges faced by PHCCs in the management of their supply chain as part of REBAHS LPSP II, challenges and facilitators of supply management of medications at PHCCs, key success and challenges in program implementation, key lessons learned, and potential for program strengthening (S5 Appendix).

#### Data analysis.

The qualitative data from the FGDs and the KIIs underwent thematic analysis using Quirkos, a qualitative analysis tool [13]. This involved systematic coding of data whereby recurring themes and patterns were identified and summarized to explore the overall impact and challenges of program implementation at the PHCC and the health system levels. The analysis also explored training and capacity-building efforts implemented along with funding mechanisms and the potential for the sustainability of the program at the health system level.

Interview transcripts and notes were coded into overarching codes (e.g., Donor role, recommendations, quality improvement plans, training and capacity building, access to services, etc.). Initial codes were reviewed by the lead researcher which allowed for the refinement and the finalization of the codes.

## Results

### Quantitative findings

Results from this analysis are divided into the (1) individual level which represents the client, and the (2) PHCC level which represents the specific services.

#### Individual level findings: Descriptive analysis.

This analysis used indicators with a mix of both absolute and relative values.

**Fig 2:**
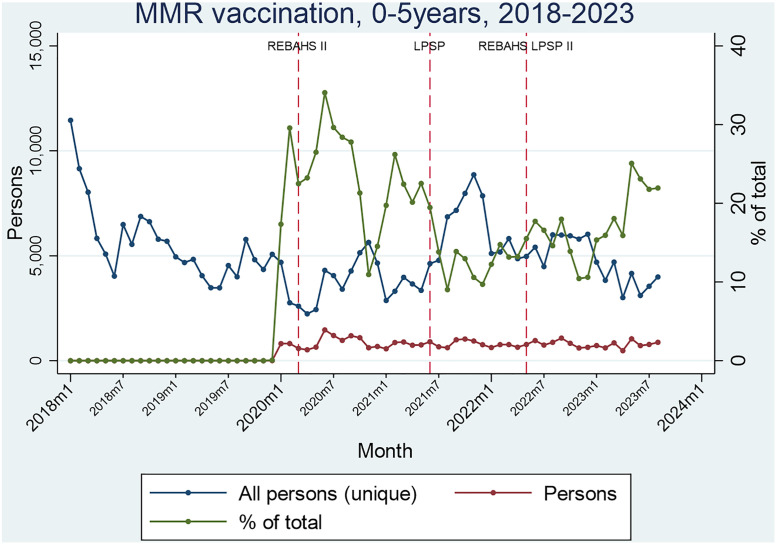
Segmented Analyses of MMR.

Measles, mumps, and rubella (MMR) vaccination services for children aged 0–5 began in 2020, peaking at the start of REBAHS II and maintaining a stable level throughout LPSP and REBAHS LPSP II. The consistency in the absolute number of MMR vaccination services indicates that they were not affected by the changes in interventions (Fig 2).

**Fig 3:**
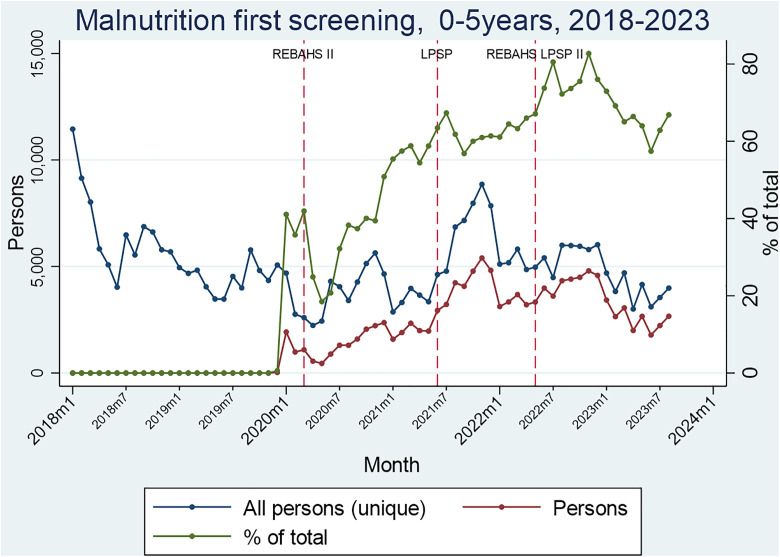
Segmented Analyses of Malnutrition.

Malnutrition screening for children aged 0–5 began in early 2020, just before REBAHS II, and steadily increased across REBAHS II and LPSP. Screening remained at a high level during REBAHS LPSP II. The proportion of eligible children screened for malnutrition increased with the onset of REBAHS LPSP II compared to previous periods (Fig 3).

Mammogram screening for breast cancer in women aged 40 and older began about four months after REBAHS LPSP II onset, with considerable variation in the number of screenings between mid-2022 and mid-2023. By August 2023, the proportion of eligible women presenting at health facilities being screened had increased, reaching nearly 40% (S6 Appendix and S7 Appendix).

**Fig 4:**
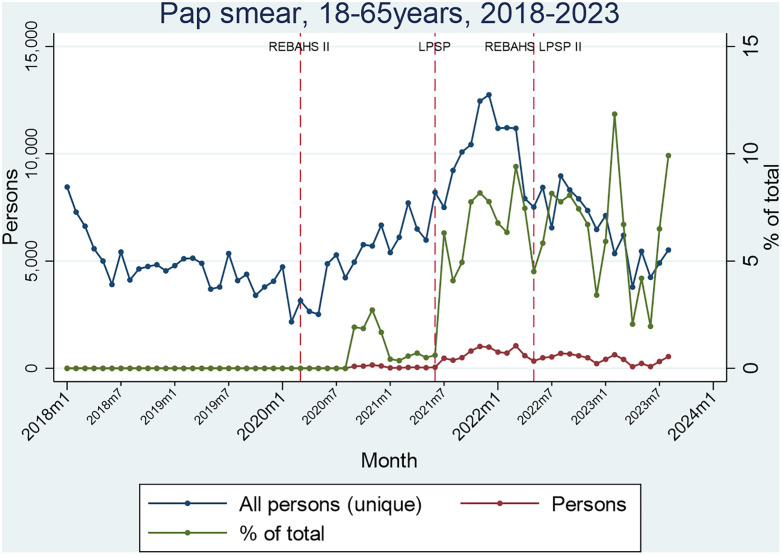
Segmented Analyses of Pap smear.

Papanicolaou (Pap smear) testing for women aged 21–65 began in mid-2020 during REBAHS II. LPSP onset was accompanied by a steep increase in tests, followed by a decline after the start of REBAHS LPSP II. The proportion of eligible women receiving the Pap test at PHCCs remained stable at about 7% throughout LPSP and REBAHS LPSP II (Fig 4).

Full fasting lipid profile testing began in the fourth month of REBAHS LPSP II, but not during REBAHS II or LPSP. About 5,000 people were tested monthly across PHCCs, with some limited variation (S6 Appendix and S7 Appendix).

**Fig 5:**
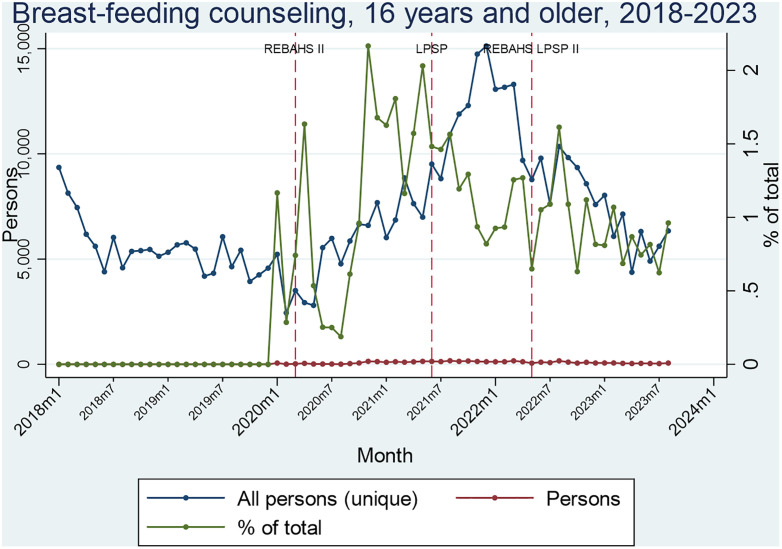
Segmented Analyses of Breastfeeding counseling.

Breastfeeding counseling began in late 2019 among women and girls aged 16 or older, with a notable increase in absolute numbers during the second half of REBAHS II, but a notable decrease during REBAHS LPSP II. The large decrease in relative figures mid-way LPSP is due to the large increase in the denominator, but was not accompanied by a similar change in absolute numbers (Fig 5, S7 Appendix).

**Fig 6:**
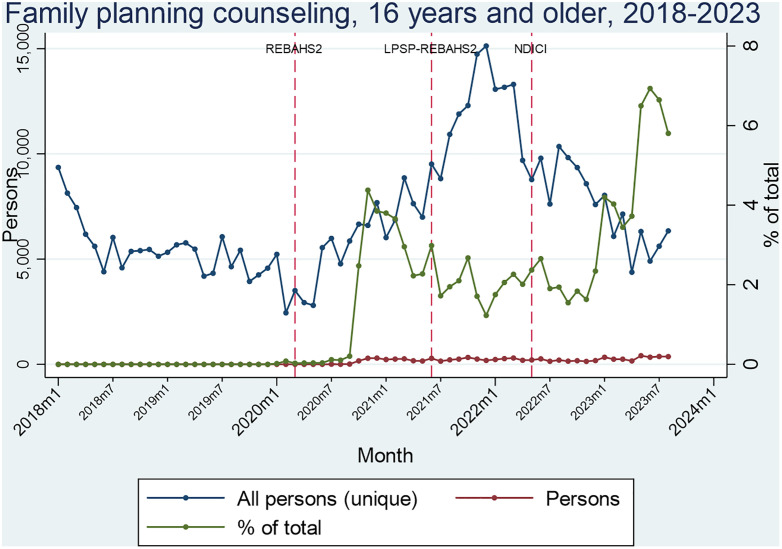
Segmented Analyses of family planning counseling.

Family planning counseling among girls and women 16 and older began halfway through LPSP, with a stable level of about 250 consultations per month across PHCCs. Eight months after the start of REBAHS LPSP II, a notable increasing trend in consultations was observed, with the proportion of girls and women counseled doubling (Fig 6).

**Fig 7:**
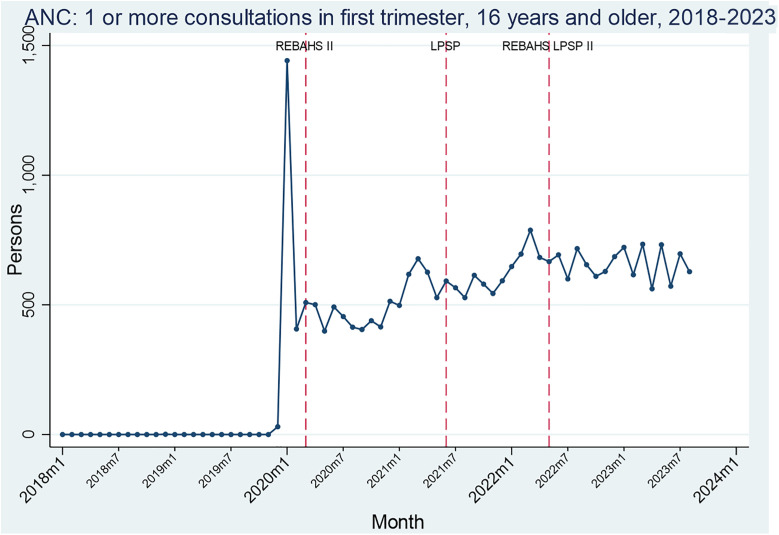
Segmented Analyses of ANC.

Antenatal care consultations (ANC) among girls and women aged 16 or older began just before REBAHS II and increased throughout the program and into LPSP. The level of consultations was maintained during REBAHS LPSP II (Fig 7).

#### Individual level findings: Interrupted time series analysis.

Time series analysis was conducted for three intervention points: one month after the start of REBAHS II, LPSP, and REBAHS LPSP II. Each period was compared to the preceding one to identify significant increases or decreases in indicator values (S5 Appendix). All significant findings are reported. Breastfeeding counseling began two months before REBAHS II, increasing by 9 service packages per month (sppm) during the program, but declined by 11 sppm during LPSP, with no statistically significant impact from REBAHS LPSP II. This decline began before REBAHS LPSP II and was not halted by its onset. For family planning counseling, REBAHS LPSP II was associated with a level decrease of 101 packages, followed by a trend shift with a 12 sppm increase. Screening for cervical cancer using a Pap smear began during REBAHS II at low levels. LPSP onset was associated with increased screening levels (+353) and trend (+74 sppm) for pap smear. REBAHS LPSP II onset was accompanied by a level decrease (-470) and a trend decrease (-80), though these declines began before REBAHS LPSP II and cannot be attributed to it. Antenatal care consultations (ANC) began three months before REBAHS II, with no change from LPSP, but a trend decrease (-19 sppm) after REBAHS LPSP II. No impact was found on malnutrition screening by LPSP or REBAHS LPSP II (S7 Appendix).

The increase in family planning counseling is closely linked to the onset of REBAHS LPSP II. Other services, including MMR vaccination, malnutrition screening, mammogram screening, fasting lipid profile testing, and gender proportions, were unsuitable for ITS analysis due to unstable trends (2018–2023) or being delivered only under REBAHS LPSP II (S7 Appendix).

#### Individual level findings: Cost-effectiveness analysis.

In 2021, Lebanon’s GDP per capita was estimated at US$4,136 (current) {Group, 2025 #19} Cost-effectiveness analysis of five services (hypertension, coronary artery disease, diabetes, antenatal care packages, and mammogram and Pap smear screenings) showed costs below the GDP per capita. This suggests these services were highly cost-effective under the REBAHS LPSP II program.

Cost-effectiveness was greatest for hypertension and CAD ($851 per DALY averted), followed by diabetes ($922), antenatal care package ($2,429), mammogram ($ 2,272), and pap smear ($ 2,657).

Sensitivity analysis assuming lower program effectiveness (half of mid-estimates) showed that all packages and services remained cost-effective, except Pap smear screening, which remained highly cost-effective. Similar conclusions applied under very low effectiveness (a third of mid-estimates). These lower scenarios are less likely, as the main analysis, based on mid-range effectiveness estimates, is considered more probable and avoids high-range assumptions (Table 3).

**Table 3:** REBAHS LPSP II program cost-effectiveness, assuming mid-range program effectiveness.

Package or service	Persons	Associated DALYs	Reduction in DALYs	Averted DALYS	Cost, package delivery	Cost per DALY averted
Hypertension & CAD	14,781	5,919	10%	592	$ 503,571	$ 851
Diabetes	9,226	2,297	20%	459	$ 423,447	$ 922
ANC	4,951	573	30%	172	$ 417,789	$ 2,429
Mammogram	7,595	213	25%	53	$ 120,840	$ 2,272
Pap smear	6,760	96	35%	34	$ 89,685	$ 2,657

#### PHCC level findings: Descriptive analysis.

**Fig 8:**
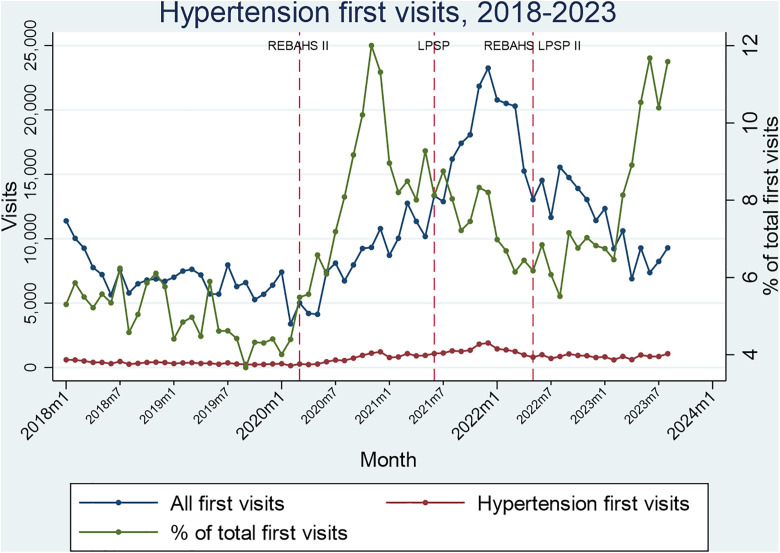
Hypertension first visits.

**Fig 9:**
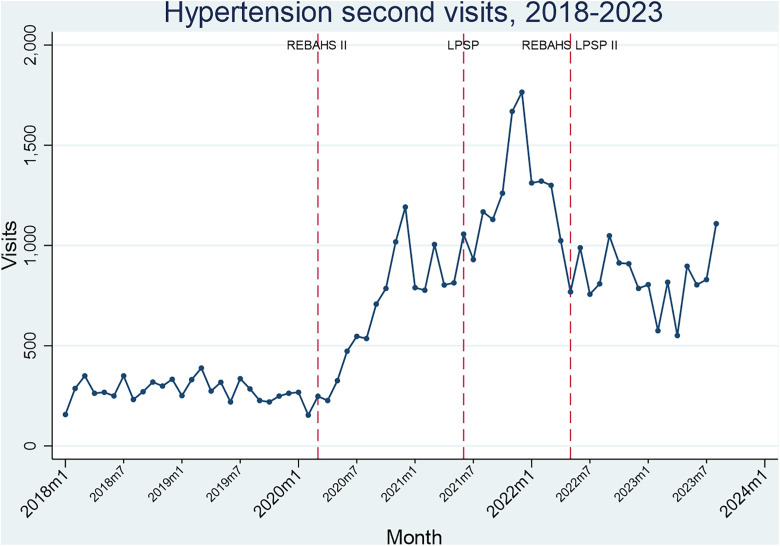
Hypertension second visits.

**Fig 10:**
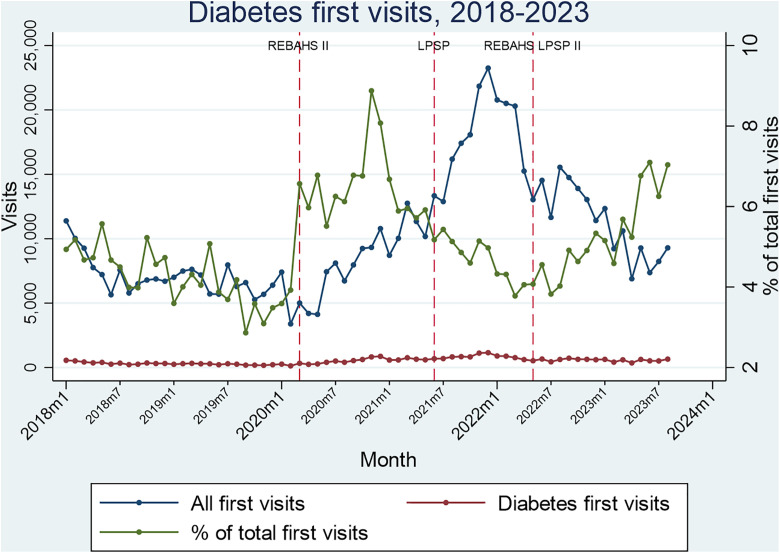
Diabetes first visits.

**Fig 11:**
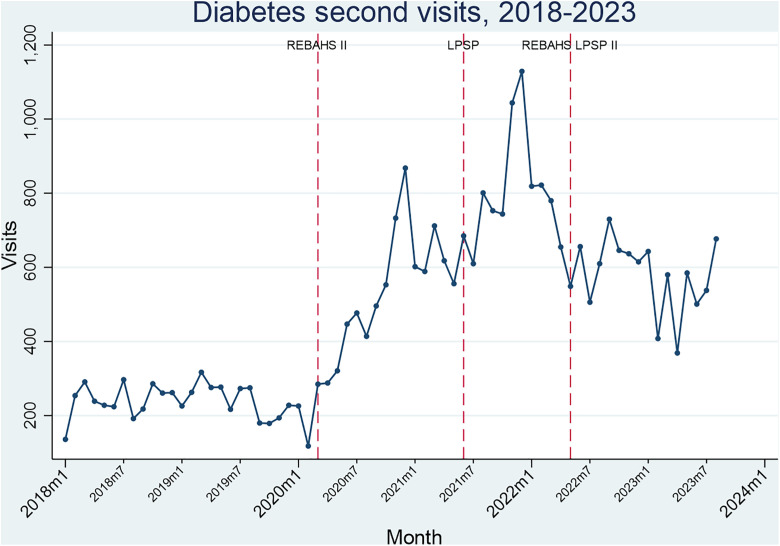
Diabetes second visits.

Hypertension and diabetes package visits rose considerably during REBAHS II and continued increasing with LPSP but began declining in early 2022 without any attributable intervention. REBAHS LPSP II was accompanied by a trend stabilization. This pattern applied to both first and second visits, indicating that the changes were driven not just by new enrollments. The number of first and second visits was very similar across all time periods which further confirms that most enrolled individuals returned to PHCCs for repeat visits (Figs 8–11).

**Fig 12:**
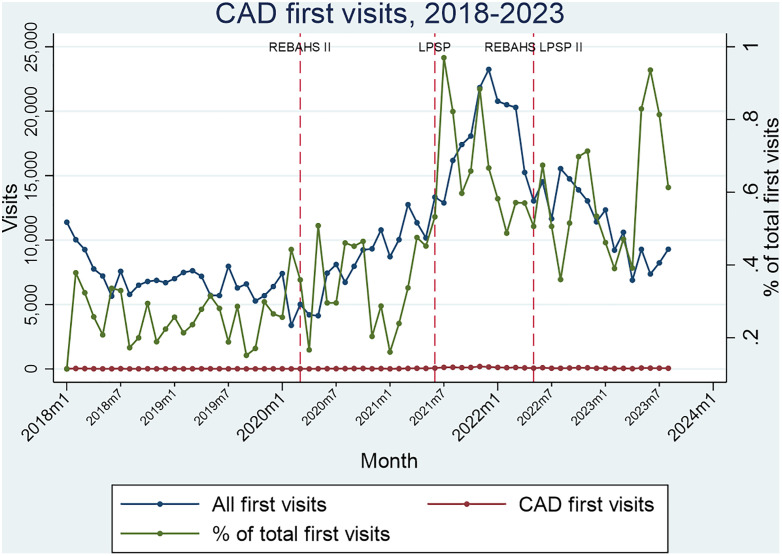
CAD first visits.

**Fig 13:**
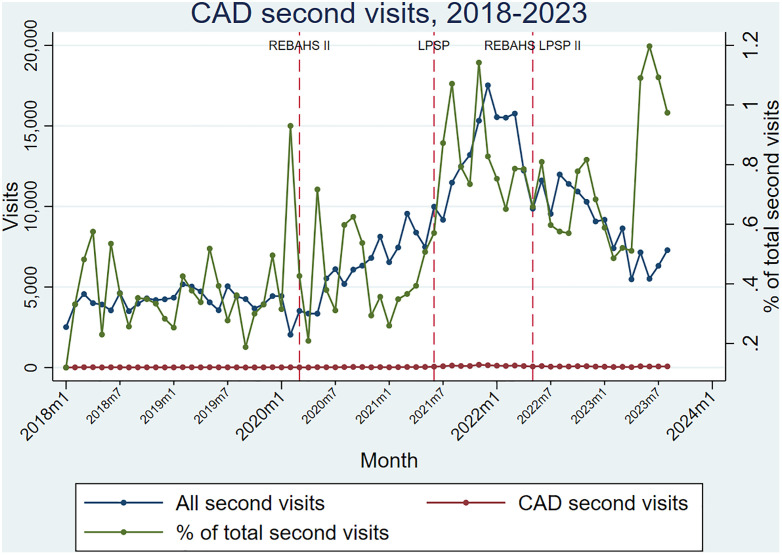
CAD second visits.

Coronary artery disease (CAD) package visits were stable at a low level during REBAHS 1 but increased by about 50% compared to all first visits during REBAHS II. LPSP onset was accompanied by a steeper rise in visits, peaking at the end of 2021. Afterward, visits declined and stabilized during REBAHS LPSP II, reaching a level about three times higher than pre-2020. These findings were consistent for both the first and second visits for CAD package services (Figs 12 and 13).

During the second half of REBAHS LPSP II, the proportion of visits for hypertension, diabetes, coronary artery disease, and chronic obstructive pulmonary disease packages increased considerably, both for the first and second visits. This rise reflects a decrease in the overall number of visits (the denominator) during REBAHS LPSP II (Figs 8–11).

**Fig 14:**
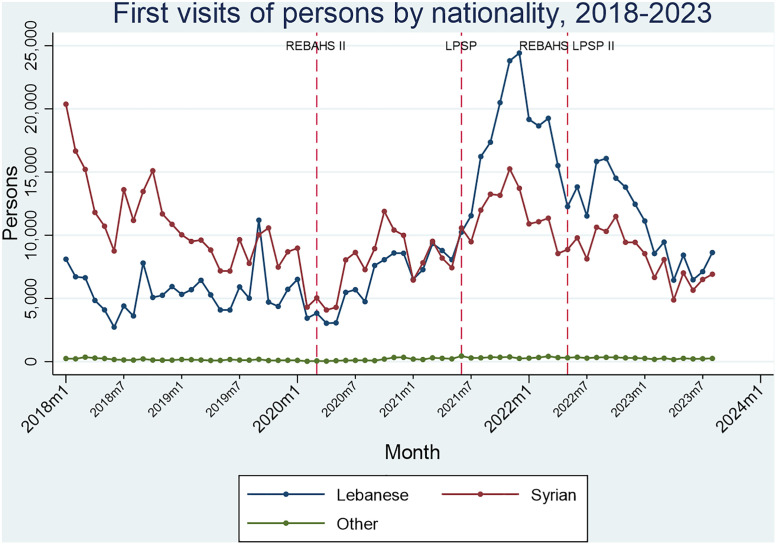
First visits of persons by nationality.

**Fig 15:**
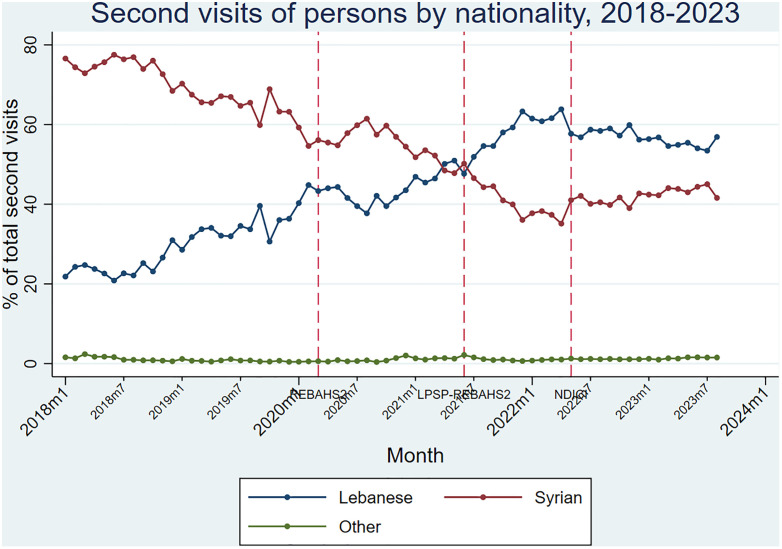
Second visits of persons by nationality.

Before REBAHS II, first visits to primary care centers were decreasing only among Syrians. With the onset of REBAHS II, first visits increased sharply for both Lebanese and Syrians, peaking halfway through LPSP. Afterward, visits declined and returned to pre-REBAHS II levels by 2023 with REBAHS LPSP II (Figs 14 and 15).

From the pre-REBAHS period through the last quarter of REBAHS II, Syrians made up the majority of first visits, followed by Lebanese, with a small proportion from other nationalities. This trend reversed at the start of LPSP, with the Lebanese becoming the majority, reflecting a pre-existing trend rather than an effect of the program. REBAHS LPSP II was associated with a decrease in the proportion of Lebanese first visits and an increase in the proportion of Syrian first visits. The gap between Lebanese and Syrian visits narrowed, especially in 2023. A similar trend was observed for second visits to PHCCs (Figs 14 and 15).

**Fig 16:**
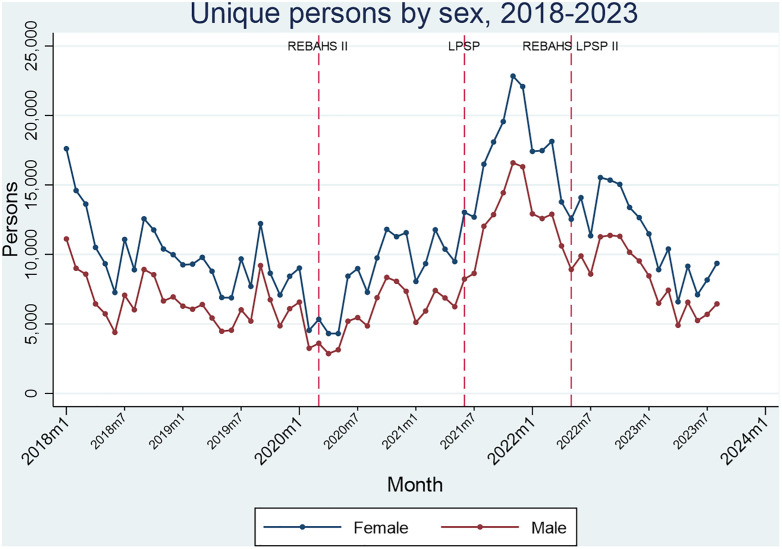
Unique persons by sex.

Throughout all three programs, more female than male persons visited PHCCs, with women and girls consistently comprising about 60% of visits from 2018–2023. The trends in primary care visits followed a similar pattern for both females and males. Visits sharply increased during REBAHS II, peaked midway through LPSP, and then decreased, stabilizing during the second half of REBAHS LPSP II in 2023 (Fig 16).

**Fig 17:**
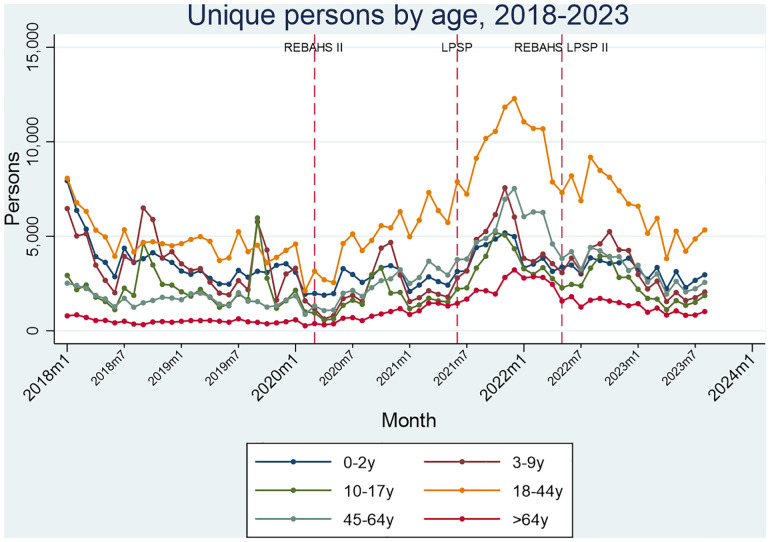
Unique persons by age.

From 2018–2023, all six age groups (0–2, 3–9, 10–17, 18–44, 45–64, and >64 years) showed a similar trend, with a sharp increase in visits during REBAHS II and a peak halfway through LPSP (Fig 17). Two notable changes in visit proportions were a long-term increase in persons aged 18–45 and those >65 years, primarily during REBAHS II and LPSP, followed by stabilization during REBAHS LPSP II. Additionally, a level decrease occurred among those >65 years after the onset of REBAHS LPSP II.

#### PHCC level findings: Interrupted time series analysis.

The start of REBAHS II was associated with a positive trend in service packages delivered for hypertension (+71 sppm), diabetes (+42 sppm), and coronary artery disease (+2 sppm), along with a level increase for diabetes packages (+169 packages). Apart from a level increase in CAD packages (+75), no significant impact of LPSP was observed among these four chronic disease packages (S8 Appendix).

The start of REBAHS LPSP II was associated with level decreases in hypertension (-565), CAD (-45), and COPD (-13) packages, but not diabetes packages. However, time series analysis suggests the program did not in itself result in a decrease in packages, as a decreasing trend preceded the program’s start. Instead, the program may have halted further reductions and stabilized package delivery levels (S8 Appendix).

The proportion of nationalities visiting PHCCs shifted between 2018–2023 among Lebanese, Syrians, and others. For Lebanese, REBAHS II onset was followed by a 0.1% monthly increase in their proportion. LPSP had no observable impact on this trend, but REBAHS LPSP II led to a level decrease (-7.9%) and a trend reversal, resulting in a 1.7% monthly decline compared to the previous period. Lebanese and Syrian proportions showed opposing patterns, as they constitute the majority of nationalities in the programs. Other nationalities, comprising 1.26% of the total, showed minimal and fluctuating changes (S8 Appendix).

Changes in primary care users’ age proportions (2018–2023) primarily occurred among the 0–2, 45–64, and >64-year groups. Among 0–2-year-olds, a sharp monthly decline of 0.68% was observed during REBAHS II, which slowed during LPSP. Just before REBAHS LPSP II, a trend reversal emerged, with a 2.53% level increase and a 0.75% monthly rise (S8 Appendix).

For those ages 45–64 years, REBAHS II onset was followed by a 0.32% monthly increase. LPSP brought a level drop (-2.84%), followed by fluctuations and a decline before stabilizing during the second half of REBAHS LPSP II (S8 Appendix).

Among those >64 years, similar trends emerged. REBAHS II led to a 0.28% monthly increase followed by a level decrease with LPSP and further declines in level and trend after REBAHS LPSP II onset (S8 Appendix).

### Qualitative results

The qualitative results of this impact evaluation are summarized according to the following overarching themes highlighting the impact, challenges, and lessons learned from the implementation of the REBAHS LPSP II initiative at both the PHCC level and the system level, in addition to recommendations for future program planning: (1) REBAHS LPSP II Program Impact, (2) REBAHS LPSP II Program Implementation Challenges, (3) Lessons Learned from the Implementation of the REBAHS LPSP II Program, (4) Recommendations for Future Program Planning. Each theme plays a critical role in shaping the future implementation of the REBAHS LPSP II program or similar initiatives.

#### REBAHS LPSP II program impact.

The REBAHS LPSP II program has been recognized for its pivotal role in *improving access to comprehensive healthcare services* as it is provided free of charge. PHCC staff members reported reduced financial barriers highlighted by an increased number of clients seeking care, specifically among those who had financial constraints. This was further highlighted by key-informants declaring an increase in the number of Lebanese individuals accessing the comprehensive healthcare services.

According to PHCC staff members, the financial support provided through the program has improved accessibility to a wider range of patients. Several services have been introduced to the PHCCs such as maternal, child, and elderly health services in addition to the provision of medications for different medical conditions (e.g., diabetes). One example includes the comprehensive package provided free of cost for women at all stages (e.g., from conception to postnatal care) through the “Safe Motherhood” package. The program was deemed particularly instrumental for the elderly population as it offers them the ability to receive comprehensive, streamlined, and cost-effective healthcare in a single location. Some PHCC staff members suggested that the program has enabled them to improve the infrastructure of their centers, further increasing the number of services provided. One notable example included the investment in medical equipment such as spirometry devices for patients with COPD. Key informants further noted a marked impact of the program on the recruitment of additional staff members at the PHCC (e.g., nurses, doctors, midwives, administrative personnel).

*“The support we got from the program, supported us in improving our services a lot. We can now invest more in other equipment and services” – PHCC staff member.*

PHCC staff members highlighted the program’s dedication to **inclusivity** as apparent in the provision of specialized facilities (e.g., physiotherapy department), home care services, and assistance for people with disabilities, which increased access to care for this population.

Most PHCC participants agreed that the program improved the *early detection and management of different medical conditions*, particularly non-communicable diseases. The inclusion of lab and radiology tests within the LPSP package has allowed for a comprehensive assessment and early diagnosis of specific conditions including hypertension, diabetes, certain cancers (e.g., vaginal and breast cancer), aneurysm, retinopathy, and child malnutrition among others. The program was reported to have enhanced the prognosis of treatment outcomes due to diligent follow-up and regular laboratory testing, facilitating the timely adjustment of treatment plans.

*“One of our patients had a mass in her breast so after we tested her, we had to request a mammography. This is an example of early detection; we can say because of these packages people are detecting their cases very early and especially the pap smears many women detected that they had cervical cancer.” – PHCC staff member*

Awareness activities implemented through the program were reported to have *increased patient awareness of available services* and the importance of preventive care. Most PHCC staff members perceived *enhanced trust in primary healthcare* among patients. This has been reflected in perceived positive changes in patient behavior, including an increase in service utilization, and a notable commitment to treatment plans and follow-ups. PHCC staff members emphasized that parents have developed *confidence in the quality of services* provided by the PHCCs.

PHCC staff members and key informants highlighted an *improvement in the quality of care at the PHCCs* through an expansion in the range of services provided, improved follow-up procedures, and an increase in the number of laboratory services available. Participants suggested that the program has improved the skills and knowledge of PHCC staff members specifically nurses and physicians, further improving the overall quality of care provided. Most PHCC staff members reported being trained on the LPSP packages including diagnostic procedures and the provision of medication. Special attention was given to the utilization of the PHENICS system, with training covering aspects such as data extraction and reporting.

*“The follow-up that happens with the patients has improved the quality of care because the patients feel they are being taken care of and are being followed up on. The service, therefore, the quality of services is being given in a better way.” - PHCC staff member*

*“The quality of services at the centers is a successful impact of the LPSP. Because due to the economic crisis, many found that PHCs were the only entry point for them and they became more confident in the comprehensive system of PHCs, the knowledge they receive from them, and the medical services they receive, even for people with disabilities for example. So, the direct impact of the REBHAS II was the provision of services and medical care to the beneficiaries among NCD patients, pregnant patients, and other patients that without the services they will not be able to afford or receive it elsewhere. – KII*

PHCC staff members have reported that the program has *improved patient satisfaction* with the services provided consequently leading to a natural referral system. PHCC staff members have heard patients share their satisfaction with the provided services, highlighting its positive impact. Patients have also shared stories about how the program has created a *ripple effect* within the community, whereby those who benefited from its services actively encouraged others to visit the PHCC and undergo essential health tests.

#### REBAHS LPSP II program implementation challenges.

Accessibility challenges, particularly transportation and financial constraints, significantly hinder healthcare access, with PHCC staff reporting that clients, especially those facing economic hardship, often struggle to afford transportation or remain unaware of nearby facilities. PHCC staff members highlighted poor patient compliance to follow-up and reluctance for preventive tests (e.g., pap smears and mammography) which have resulted in delayed diagnosis and treatment. Staff members highlighted administrative burdens, including excessive documentation and gaps in the national health information system of PHCCs posing challenges regarding the efficient implementation and monitoring of the program implementation. It was suggested that financial and human resource limitations, compounded by economic challenges and high turnover rates among healthcare professionals, affected the quality and continuity of services. Rising operational costs and funding uncertainties have raised concerns relevant to the sustainability of services beyond funding provided to the current program. Staff members from PHCCs expressed fears of potential discontinuation of the program if funding is omitted, positing that such an event would impede the centers’ ability to maintain the quality or quantity of provided services and meet salary obligations.

*“This is a major problem that we are facing, for example, we were able to catch and convince a lot of women to do their mammography which is for free now. If the program stops, we can’t afford to do that and we will lose our patients. We are working according to our available resources. Also, we added a lot of human resources due to this program. If the program stops, we will not be able to afford to pay the salaries.” – PHCC staff member*

#### Lessons learned and recommendations from the implementation of the REBAHS LPSP II program.

Key lessons learned at the PHCC level highlight the importance of tailoring healthcare services to individual patient needs and adopting a holistic approach that integrates treatment, prevention, education, and community involvement. Recognizing that each patient requires specialized care, PHCC staff members acquired critical skills in distinguishing between cases and providing tailored care and communication to patients. PHCC staff members recognized the indispensable role of community engagement in shaping effective and meaningful patient outcomes. By actively involving the community, healthcare providers gain insights into the distinctive needs, preferences, and challenges of the local population. Additionally, the program underscored the need for infrastructure expansion to accommodate a higher patient load and enhance service delivery.

Key lessons learned at the system level emphasized the importance of identifying priorities, aligning efforts across relevant stakeholders, and engaging with the MoPH, donor agencies, and national NGOs to streamline processes and prevent service overlap, optimizing coverage. Regular meetings and dialogues across PHCCs about program implementation and new initiatives helped in addressing challenges and fostering a more responsive healthcare system. Capacity building for PHCC staff emerged as a cornerstone for sustainability, with training on the utilization of the national PHCC health information system for documentation as critical. Flexibility and adaptability were also highlighted as key enablers of success, to achieve project goals and remain flexible and adapt to evolving circumstances.

## Discussion

The REBAHS LPSP II project has brought forth several prominent impacts that have significantly shaped the landscape of healthcare delivery among PHCCs in Lebanon. A key outcome includes amplified access and affordability of healthcare services reflected by a substantial increase in the number of patients utilizing the PHCC services. This is consistent with the findings in Afghanistan whereby the introduction of a Benefit Package of Health Services led to an increase in utilization with the number of patients visiting the PHCCs increasing from 450 per month in 1973–2209 in 2011 [14]. The introduction of comprehensive screening packages has facilitated the early detection of diseases such as diabetes mellitus and has streamlined the management and treatment protocols of such conditions. In line with the literature comprehensive health check-ups in PHCCs (e.g., vital signs, family history of diseases, laboratory tests for blood, urine and stools, social history, mental health history, cancer screening assessment) complemented by counseling, referrals, medical investigations and follow-ups, health education, and medication prescription where needed lead to early detection and promote early intervention and management for patients with non-communicable diseases [15]. The establishment of integrated care teams (ICT) (e.g., multidisciplinary practitioners working together to deliver comprehensive, multifaceted care to patients) within the Singaporean primary healthcare system has resulted in improved health and process outcomes for patients with multimorbidity (e.g., hypertension, and/or type 2 diabetes mellitus, and/or hyperlipidemia) [16]. Patients treated by ICT were more likely to attain their treatment goal after 12 months, and attend their annual diabetic foot and eye screening tests when applicable [16].

At the individual level, the program implementation resulted in an overall increase in the proportion of children visiting the PHCC and being screened for malnutrition, women visiting the facility and being screened for breast cancer via mammogram testing, and a high number of antenatal care visits. Nevertheless, a decreased trend was observed for some other services including breastfeeding counseling, and pap smear testing which may have been attributed to the confounding variables, rather than the program itself. The decrease in pap smear testing could be due to the fact that most women had already benefited from the service before the initiation of the program and thus were no longer eligible for testing. The decrease in breastfeeding counseling may be linked to a decrease in ANC consultation during this period due to external factors including the COVID-19 pandemic and a limited number of midwives trained in infant and young child feeding counseling at the PHCCs.

At the primary healthcare level, various changes in the level or trend of different services have been associated with the REBAHS II, LPSP, and REBAHS LPSP II programs, often increasing services. For REBAHS LPSP II, ITS analysis shows some changes began before REBAHS LPSP II, with services peaking before LPSP ended, followed by declines that REBAHS LPSP II stabilized. Therefore, some decreases cannot be attributed solely to REBAHS LPSP II, as the absence of the program may have led to further declines. However, specific impacts linked to REBAHS LPSP II include increased family planning counseling, a shift in nationality proportions (more Syrians, fewer Lebanese), a rise in children aged 0–2 years, and declines in adults aged 45–64 years and >64 years (S8 Appendix).

The WHO Global Disability Action Plan 2014–2021 emphasizes the importance of integrating rehabilitation services for people with disabilities across all levels of care including primary healthcare to achieve improved functioning and quality of life for this population [17]. The REBAHS LPSP II program has made significant progress in promoting inclusivity, particularly for people with disabilities. The provision of rehabilitation services, such as physiotherapy and wheelchair-accessible facilities, has played a pivotal role in enhancing accessibility while accommodating the services for individuals with disabilities.

Challenges faced in the implementation of the REBAHS LPSP II project are multifaceted and are aligned with common barriers recognized in the implementation of Essential Health Packages globally [4]. Resource constraints such as medication procurement and delivery delays have impacted the timely provision of medications to patients [18]. Financial constraints are exacerbated by the ongoing economic crisis in Lebanon, affecting the sustainability of services in PHCCs, and leading to staff reimbursement issues. Transportation cost has emerged as a key barrier to accessibility [4,18,19]. Other noteworthy challenges that were highlighted as part of this study include the rigidity of the packages implemented and the saturation of certain specialties as a result of a large load of patients coupled with an inefficient referral system from the general practitioners to the specialized physicians [20].

Two key lessons have emerged from the implementation of the program in line with global literature. Communication and collaboration emerged as pivotal elements throughout the project’s lifecycle. Identifying priorities, aligning efforts, and engaging with key stakeholders, including the MoPH, was central to achieving optimal outcomes of the program, which emphasized the need for transparent communication, within the project framework and with external partners and policymakers [4].

Key recommendations have emerged from the program implementation to improve the healthcare system. Promoting inclusive healthcare access is imperative for ensuring that vulnerable and underserved populations receive the necessary medical care. Initiatives aimed at addressing the gap in access for people with disabilities, as well as expanding services to accommodate the diverse needs of the community, should be prioritized. This recommendation emphasizes the commitment to equitable healthcare, acknowledging the importance of reaching every segment of the population. Enhancing human resources emerges as a critical recommendation, highlighting the need for a robust and well-trained healthcare workforce [21]. Investing in the recruitment, training, and retention of healthcare professionals, including nurses and doctors, is essential for overcoming challenges related to the shortage of personnel [20,21]. Strengthening communication and collaboration with the MoPH, stakeholders, and the broader healthcare community is identified as a key factor in optimizing healthcare outcomes [4].

## Limitation

This evaluation highlighted limitations to the evaluations due to limited documentation of some information. It was not possible to identify underreported services using the PHENICS database for PHCCs in Lebanon. Data analysis was conducted using all relevant available data. Although missing data from previously non-reporting PHCCs was expected, the level of missing data for many general indicators (e.g., packages delivered) was considered to be minimal among PHCCs continuing to report on indicators. The level of missing data for more specific variables (e.g., blood tests conducted) was expected to be greater and therefore less reliable. Assessment of all PHCC visits between 2018 and 2023 found that only 3 out of 64 PHCCs ceased reporting or delivering services during this period (comprising less than 1% of all visits), which supports the assumption of a low level of missing data at the general indicators, and strengthens the conclusions reached.

A minor limitation encountered while measuring the DALYs at the PHCC level includes the approach. The age breakdown from the WHO DALYs includes 0–4 years, 5–14 years, 15–29 years, and then ten-year categories up to +70 years. The first three categories did not fully correspond with the PHCC services delivered by age group; however, this was expected to have minimal effect on the final cost-effectiveness figures developed. An additional limitation of the CEA is the assumption of program sustainability, although the sensitivity analysis reflect decreased services and/or effectiveness, using one-half and one-third of the program effectiveness used for the main results.

Interrupted time series analysis further relies on an assumption that changes from one period to another are due to the intervention or, in this case, the implementation of different stages of the program. However, external factors were likely to have affected both levels and trends over time, especially given the long study period.

Furthermore, during the study period, the population in catchment areas of PHCCs was not stable. As a result, we used the volume of relevant clients using services at PHCCs as the denominator for the calculations of proportion. As a result, we are unable to extend the findings to the population level; rather, they are limited to the population of healthcare-seeking individuals presenting at PHCCs.

## Conclusion

The REBAHS LPSP II program was shown to be promising in leaving a positive imprint on Lebanon’s healthcare landscape, notably marked by enhanced accessibility and affordability of healthcare services, and improved service quality. These have been reflected by an increase in service utilization, early detection, and tailored treatment protocols, which have led to better patient health outcomes. However, operational, financial, and structural challenges persist, hindering the project’s full effectiveness. This study may serve as a model for improving healthcare systems at the national, regional, and international levels, offering valuable insights into optimizing resource allocation, improving global funding models, and amplifying program impact. Ultimately this study may serve as a model for improving healthcare access, health service delivery, and sustainability, particularly in conflict-affected settings and countries with limited resources.

## Supporting information

S1 Appendix Services included in the different LPSP packages. (PDF)

S2 Appendix Definition of key terms for interrupted time series analysis. (PDF)

S3 Appendix PHC management and staff interview guide. (PDF)

S4 Appendix Donors/INGOs interview guide. (PDF)

S5 Appendix Ministry of Public Health interview guide. (PDF)

S6 Appendix Segmented analyses of mammogram screening and full fasting lipid profile. (PDF)

S7 Appendix Interrupted time series analysis for malnutrition screening, pep smear screening, breastfeeding counselling, family planning counselling, antenatal care consultations. (PDF)

S8 Appendix Interrupted time series analysis for hypertension package, diabetes package, coronary artery disease package, COPD package, nationality Lebanese, ages 0–2 years, proportion, ages 3–9 years, proportion, ages 45–64 years, proportion, ages ≥65 years, proportion. (PDF)
