# Supplementary material for: Mixed method program impact evaluation: Reducing economic barriers to accessing health services (REBAHS) long-term primary healthcare subsidization protocol (LPSP) II action in Lebanon
Source: PLOS Glob Public Health. 2025 Dec 5;5(12):e0005569. doi: 10.1371/journal.pgph.0005569 (PMC12680163; doi:10.1371/journal.pgph.0005569)
Supplement: S1 Appendix — (PDF) [file pgph.0005569.s001.pdf]

S1 Appendix. Services Included in the Different LPSP Packages

Service		Health and Wellness for children and adolescents (18 years old and under)	Health and Wellbeing for Women (19-64 years)	Health and wellness for males (19-64 years)
**Immunization**	The package offers the following vaccines:	• Hepatitis B<br>• Penta (DPT, Hib, Hepatitis B)<br>• IPV<br>• OPV<br>• PCV 13<br>• Measles<br>• MMR<br>• DPT<br>• Td	Td	Td
**Laboratory**	The package offers the following laboratory testing:		• Lipid Profile<br>• Blood Sugar (F)<br>• CBC<br>• Urine analysis (dipstick)<br>• HIV (VCT)<br>• FIT Test	• Lipid Profile<br>• Blood Sugar (F)<br>• CBC<br>• Urine analysis (dipstick)<br>• HIV (VCT)<br>• FIT Test
**Radiology**	The package offers the following imaging radiology services:		Mammography	
**Other Tests**	The package offers the following tests:		Pap smear	
**Screening tests**	The package offers the following screening tests:	CBC, platelets		
**Consultations**	During a consultation with a healthcare professional,	• Height<br>• Weight<br>• BMI Calculation<br>• Head Circumference	• Height<br>• Weight<br>• BMI Calculation<br>• Blood Pressure	• Height<br>• Weight<br>• BMI Calculation<br>• Blood Pressure

	the package offers clinical assessments for the following:	• Blood Pressure<br>• Malnutrition Assessment<br>• Signs of Abuse Child<br>• Development<br>• Developmental Surveillance<br>• Vision screening<br>• Assess Hearing risk.<br>• Screen for eating disorders.<br>• Screen for depression<br>• Screen for risky health behaviors (substance misuse, suicide, violence, others)	• Depression PQH2<br>• Signs of Abuse<br>• CVD Assessment<br>• FRAX	• Depression PQH2<br>• Signs of Abuse<br>• CVD Assessment
**Counseling**	The package offers the following counseling initiatives:	• Oral Health<br>• Infant & Child Feeding Counseling<br>• Diet Advice<br>• Injury Prevention<br>• Passive Smoking /Smoking<br>• Exercise<br>• Abuse (domestic, bullying, GBV, child marriage, others)<br>• Puberty (self-care, menstrual hygiene management)<br>• Substance abuse (including drugs and alcohol)<br>• Reproductive & Sexual Health	• Smoking<br>• Diet Advice<br>• Exercise Advice<br>• Substance abuse (including drugs, alcohol)<br>• Abuse (domestic, intimate partner violence, gender-based violence, other)<br>• Reproductive & Sexual Health Counseling (HIV, STI & reproductive tract infections, sexual disorders, Infertility, Pregnancy Prevention, post-abortion care, others)<br>• Menopause	• Smoking<br>• Diet Advice<br>• Exercise Advice<br>• Substance abuse (including drugs, alcohol)<br>• Abuse (domestic, intimate partner violence, gender-based violence, other)<br>• Reproductive & Sexual Health Counseling (HIV, STI & reproductive tract infections, sexual disorders, Infertility, Pregnancy Prevention,

		Counseling (HIV, STI & reproductive tract infections & Pregnancy Prevention)		post-abortion care, others)<br>• Andropause
**Medications (as needed)**	The package offers the following medications:	All essential medications	All essential medications	Essential Medications as needed
**Family Planning**	The package offers the following family planning services:		• Family Planning<br>• Visit Counseling about Family Planning<br>• Preconception counseling<br>• Modern Contraception method<br>• Couple Counseling	

**Service**		***Health and wellness for the elderly (65 years and above)***	***Non-communicable diseases - type II diabetes***	***Non-communicable diseases - high blood pressure***
**Healthcare Professionals Consultations**	**The following healthcare professionals can be consulted as part of the package:**		• **Family Doctor**<br>• **Ophthalmologist**<br>• **Clinical Dietician (if available)**	• **Family Doctor**<br>• **Ophthalmologist**<br>• **Clinical Dietician (if available)**
**Immunization**	**The package offers the following vaccines:**	• **Td**<br>• **Flu Vaccine**<br>• **Pneumococcal**	• **Flu Vaccine**<br>• **Hepatitis B (if not taken)**	**Flu Vaccine**

			• Pneumococcal PPSV 23 (if not taken)	
**Laboratory**	**The package offers the following laboratory testing:**	• **Lipid Profile**<br>• **Blood Sugar (F)**<br>• **CBC**<br>• **Urine analysis (dipstick)**<br>• **FIT Test**	• **Blood Sugar (F)**<br>• **Hba1c**<br>• **Lipid Profile**<br>• **Creatinine**<br>• **SGPT**<br>• **SGOT**<br>• **CBC, platelets**<br>• **Spot Urine for Microalbumin**	• **Blood Sugar (F)**<br>• **Lipid Profile**<br>• **Sodium**<br>• **Potassium**<br>• **Creatinine**<br>• **Uric Acid**<br>• **Calcium**<br>• **CBC, Platelets**<br>• **Urinalysis**<br>• **Spot Urine for Microalbumin**
**Radiology**	**The package offers the following imaging radiology services:**	**Mammography (women)**		**EKG**
**Other Tests**	**The package offers the following tests:**	**Pap smear (women)**	**EKG**	
**Consultations**	**During a consultation with a healthcare professional, the package offers clinical assessments for the following:**	• **Height**<br>• **Weight**<br>• **BMI Calculation**<br>• **Blood Pressure**<br>• **Depression PQH2**<br>• **CVD Assessment**	• **Height**<br>• **Weight**<br>• **Blood Pressure**<br>• **Foot Exam**<br>• **Depression PHQ2**<br>• **CVD Assessment**	• **Height**<br>• **Weight**<br>• **Blood Pressure**<br>• **Foot Exam**<br>• **Depression PHQ2**<br>• **CVD Assessment**

		• Mini Mental Test<br>• Activities of Daily Living<br>• Gait and Balance, Vision Screening<br>• Hearing Screening	• Medications Reconciliation	• Medications Reconciliation
**Counseling**	**The package offers the following counseling initiatives:**	• Smoking<br>• Diet Advice<br>• Exercise Advice<br>• Alcohol Urinary<br>• Incontinence<br>• Fall Prevention<br>• Polypharmacy<br>• Social (Elder) abuse & neglect<br>• Reproductive & Sexual Health Counseling (STI, sexual disorders, others)	• Smoking<br>• Diet Advice<br>• Exercise Advice<br>• Care Plan<br>• Diabetes Self-Management Education	• Smoking<br>• Diet Advice<br>• Exercise Advice<br>• Care Plan<br>• Hypertension Self-Management Education
**Medications**	**The package offers the following medications:**	**Essential Medications as needed**	**Medication based on Chronic medication list provided by MoPH**	**Medication based on Chronic medication list provided by MoPH**

**Service**	**Non-communicable diseases - coronary heart disease**	**Non-communicable diseases – chronic obstructive pulmonary disease**	**Safe motherhood**

**Health Care Professionals Consultations**	**The following healthcare professionals can be consulted as part of the package:**	• **Family Doctor**<br>• **Clinical Dietician (if available)**	• **Family Doctor**<br>• **Clinical Dietician (if available)**	• **Obstetrician**<br>• **Midwife**
**Immunization**	**The package offers the following vaccines:**	• **Flu Vaccine**<br>• **Pneumococcal PPSV 23 (if not taken)**	• **Flu Vaccine**<br>• **Pneumococcal PPSV 23 (if not taken)**	
**Laboratory**	**The package offers the following laboratory testing:**	• **Blood Sugar (F)**<br>• **Lipid Profile**		• **CBC, platelets**<br>• **Urinalysis**<br>• **Blood Group & Rhesus (done if not known before)**<br>• **Rubella Titter (IgG)**<br>• **Toxoplasma Titter (IgG &IgM)**<br>• **Hepatitis B (HBSAg)**<br>• **HIV**<br>• **Syphilis (VDRL)**<br>• **Urine Culture**<br>• **1h post glucose challenge test**<br>• **Indirect Coombs**
**Other Tests**	**The package offers the following tests:**	• **EKG**<br>• **Echocardiography**	• **Pulmonary Function Test**<br>• **CXR**	• **Ultrasound**<br>• **Urine Dipstick/urinalysis if dipstick not available (done in lab)- every visit**

**Consultations**	**During a consultation with a healthcare professional, the package offers clinical assessments for the following:**	• **Height**<br>• **Weight**<br>• **Blood Pressure**<br>• **Depression PQH2**<br>• **Medication Reconciliation**	• **Height**<br>• **Weight**<br>• **Blood Pressure**<br>• **Depression PQH2**<br>• **COPD Assessment (GOLD/CAT Score)**<br>• **Medication Reconciliation**	• **Height**<br>• **Weight**<br>• **BMI**<br>• **Blood Pressure**<br>• **Fundal Height**<br>• **Fetal Heart Rate**<br>• **Peripheral edema**<br>• **Abdominal Palpation-Fetal Presentation**
**Counseling**	**The package offers the following counseling initiatives:**	• **Smoking (smoking cessation program)**<br>• **Diet Advice**<br>• **Exercise Advice**<br>• **Care Plan**<br>• **CAD Self-Management Education**	• **Smoking (smoking cessation program)**<br>• **Diet Advice**<br>• **Alcohol**<br>• **Exercise Advice**<br>• **Care Plan**<br>• **COPD Self-Management Education**	• **Normal Changes of Pregnancy**<br>• **Nutrition**<br>• **Exercise**<br>• **Tobacco & Substance Abuse**<br>• **Alcohol**<br>• **Medication & Herbal products**<br>• **Sexual Activity**<br>• **Heartburn**<br>• **Screening for Depression**<br>• **Screening for Gender-based violence, intimate partner violence**<br>• **Breastfeeding**
**Medications**	**The package offers the**	**Medication based on Chronic medication**	**Medication based on Chronic**	• **Folic Acid**

	following medications:	list provided by MoPH	medication list provided by MoPH	• Iron<br>• Calcium<br>• Vitamin D<br>• Rhogam
				**Post-Partum**
				**Family Planning**
