# Supplementary material for: Mixed method program impact evaluation: Reducing economic barriers to accessing health services (REBAHS) long-term primary healthcare subsidization protocol (LPSP) II action in Lebanon
Source: PLOS Glob Public Health. 2025 Dec 5;5(12):e0005569. doi: 10.1371/journal.pgph.0005569 (PMC12680163; doi:10.1371/journal.pgph.0005569)
Supplement: S2 Appendix — (PDF) [file pgph.0005569.s002.pdf]

S2 Appendix. Definition of key terms for interrupted time series analysis.

1. **Trend**: This refers to the general direction in which the data is moving over time. In time series analysis, trends can be upward, downward, or stable. The trend can be assessed using various statistical methods, such as regression analysis, to identify patterns in the data over a specified period (Kontopantelis, et al., 2015; Cruz, et al., 2019).
2. **Pre-Interruption/ pre-intervention Trend**: This is the trend observed in the time series data before the interruption occurs. It represents the underlying pattern of the data prior to any intervention or event that may cause a change in the series. Analyzing the pre-interruption trend helps establish a baseline for comparison with the post-interruption data (Kontopantelis, et al., 2015; Cruz, et al., 2019).
3. **Interruption Point**: This is the specific point in time at which an intervention or event occurs that is expected to affect the time series data. The interruption point is critical for analyzing the impact of the intervention, as it serves as the dividing line between the pre-interruption and post-interruption periods (Kontopantelis, et al., 2015; Cruz, et al., 2019).
4. **Change in Slope**: This term describes the alteration in the rate of change of the time series data after the interruption point. It indicates whether the trend has become steeper (increased slope), flatter (decreased slope), or reversed (negative slope) following the intervention. This change in slope helps to understand how the intervention has affected the ongoing trend of the data (Kontopantelis, et al., 2015; Cruz, et al., 2019).
5. **Post-Interruption/post-intervention Trend**: This is the trend observed in the time series data after the interruption has occurred. It reflects how the data behaves following the intervention and is compared to the pre-interruption trend to assess the impact of the intervention over time (Kontopantelis, et al., 2015; Cruz, et al., 2019).
