# Supplementary material for: Mixed method program impact evaluation: Reducing economic barriers to accessing health services (REBAHS) long-term primary healthcare subsidization protocol (LPSP) II action in Lebanon
Source: PLOS Glob Public Health. 2025 Dec 5;5(12):e0005569. doi: 10.1371/journal.pgph.0005569 (PMC12680163; doi:10.1371/journal.pgph.0005569)
Supplement: S3 Appendix — (PDF) [file pgph.0005569.s003.pdf]

S3 Appendix. PHC Management and Staff Interview Guide.

1) What services are offered at the PHCC?

2) Since the implementation of REBAHS-LSPS-II, how has the center adapted to improve equitable access to comprehensive quality of care?

3) How has the implementation of REBAHS-LPSP-II improved access to care?

Probe: how about people with disabilities? Access to laboratory services?

4) What is the perceived impact of the LPSP packages on the community?

Probe: according to each package; (1) health and Wellness for children and adolescents (18 years old and under), (2) Health and Wellbeing for Women (19-64 years), (3)Health and wellness for males (19-64 years), (4)Health and wellness for the elderly (65 years and above), (5)Non-communicable diseases - type II diabetes, (6)Non-communicable diseases - high blood pressure, (7)Non-communicable diseases - coronary heart disease, (8) Non-communicable diseases – chronic obstructive pulmonary disease, (9)Safe motherhood

Probe: are there examples where the utilization of wellness packages has led to the early detection or prevention of health issues among beneficiaries?

5) What are some examples of successful outcomes achieved by the primary healthcare centers through the REBAHS-LPSP II program?

Probe: according to each package; (1) health and Wellness for children and adolescents (18 years old and under), (2) Health and Wellbeing for Women (19-64 years), (3)Health and wellness for males (19-64 years), (4)Health and wellness for the elderly (65 years and above), (5)Non-communicable diseases - type II diabetes, (6)Non-communicable diseases - high blood pressure, (7)Non-communicable diseases - coronary heart disease, (8) Non-communicable diseases – chronic obstructive pulmonary disease, (9)Safe motherhood

6) What methods have been employed to effectively communicate the importance and benefits of wellness packages to the community?

7) How can the REBAHS-LPSP-II program be strengthened to improve access and quality of care at PHCs?

8) How have quality improvement plans been integrated into the implementation of LPSP packages at the PHC level?

9) What approaches have been adopted for capacity building of PHC staff to effectively deliver the LPSP packages?

10) What are the key lessons learned from the implementation of the REBAHS-LPSP-II program Probe: how might these lessons be applied to similar programs in the future?

11) How can services under the LPSP packages can be improved even further?

12) What is the center's future plans or strategies for sustaining and further improving the primary healthcare centers' operations beyond the REBAHS-LPSP II program?

Probe: how can the gains be sustained?

Health Systems Strengthening

13) What strategies and practices are employed by the center to manage and monitor medication supplies and supplies of medical equipment?

Probe: What measures are in place to address any issues that arise?

14) Have there been any capacity-building initiatives for the staff working at the primary healthcare centers as part to REBAHS-LPSP II? If yes, how has this affected service delivery?

15) What are some of the challenges faced when it comes to the implementation of the REBAHS-LPSP-II?

Probe: Access to medication, supply of medical supplies, consultations financing etc...

16) How can the supply-chain management (i.e., medication supplies, equipment supplies, etc.) be improved for the PHC?

17) Since the establishment of the REBAHS II, has there been any change in the referral system between PHCs and higher-level healthcare facilities?

Probe: What protocols or systems are in place to facilitate referrals? What were the challenges?
