# Supplementary material for: Mixed method program impact evaluation: Reducing economic barriers to accessing health services (REBAHS) long-term primary healthcare subsidization protocol (LPSP) II action in Lebanon
Source: PLOS Glob Public Health. 2025 Dec 5;5(12):e0005569. doi: 10.1371/journal.pgph.0005569 (PMC12680163; doi:10.1371/journal.pgph.0005569)
Supplement: S4 Appendix — (PDF) [file pgph.0005569.s004.pdf]

S4 Appendix. Donors/INGOs Interview Guide.

1) How are primary healthcare centers engaged in program design activities, including needs assessment, stakeholder involvement, and evidence-based decision making?
2) When evaluating the primary activities (program design, fund management, and program delivery) within the value chain, what criteria is used to assess their alignment with the overall objectives of primary healthcare centers and the communities they serve?
3) What are the key challenges and barriers faced by the primary healthcare center in optimizing its value chain?
4) How do donor agencies prioritize and allocate funds to different projects or initiatives?
    a. Probe: What factors influence their decision-making process?
    b. Probe: What are the major challenges and opportunities in fund management for primary healthcare centers?
    c. Probe: How can financial practices be optimized to ensure adequate funding, transparency, and accountability in the delivery of healthcare services?
5) What data collection and monitoring systems are in place to assess the impact and effectiveness of the primary healthcare centers' interventions?
6) How do the donors, INGO, and local NGO collaborate and coordinate their efforts to support the primary healthcare centers under the REBAHS-LPSP II program?
7) What strategies are in place to ensure sustainability of programs?
8) How can INGOs and donors contribute to strengthening the value chain and primary activities, such as program design, fund management, and program delivery, to ensure greater effectiveness and impact in the overall implementation of the Action initiative?
9) How can INGOs and donors work collaboratively with primary healthcare centers to build local capacity, foster knowledge exchange, and enhance skill development among healthcare personnel and administrators?
10) How can INGOs and donors support primary healthcare centers in overcoming barriers to program delivery and ensure equitable access to healthcare services for vulnerable populations, particularly in remote or underserved areas?

11) What were key successes of the REBAHS-LPSP-II?
12) What are some of the challenges faced when it comes to the implementation of the REBAHS-LPSP-II?
13) How can the REBAHS-LPSP-II program be strengthened to improve access and quality of care at PHCs?
14) What are the key lessons learned from the implementation of the REBAHS-LPSP-II program?

    Probe: how might these lessons be applied to similar programs in the future?
