# Supplementary material for: Mixed method program impact evaluation: Reducing economic barriers to accessing health services (REBAHS) long-term primary healthcare subsidization protocol (LPSP) II action in Lebanon
Source: PLOS Glob Public Health. 2025 Dec 5;5(12):e0005569. doi: 10.1371/journal.pgph.0005569 (PMC12680163; doi:10.1371/journal.pgph.0005569)
Supplement: S5 Appendix — (PDF) [file pgph.0005569.s005.pdf]

S5 Appendix. Ministry of Public Health Interview Guide.

1) How does the Ministry of Public Health collaborate with relevant stakeholders, including donors and INGOs, to strengthen the cross-cutting support activities within the value chain (i.e., management, human resource management, information technology, finance, and supply-chain management) for primary healthcare centers as part of the REBAHS-LPSP II program?
2) What are the key challenges faced by primary healthcare facilities in effectively managing their supply chains as part of the REBAHS-LPSP II?
    a. Probe: how do these challenges impact the quality and accessibility of healthcare services?
    b. Probe: How does the Ministry of Public Health address the challenges related to supply-chain management for primary healthcare centers?
3) What are some challenges in acquiring medical supplies and medications for PHC?
    a. Probe: How can those challenges be overcome?
4) What are some facilitators that could improve supply management of medications at the PHC?
5) What were key successes of the REBAHS-LPSP-II?
6) What are some of the challenges faced when it comes to the implementation of the REBAHS-LPSP-II?
7) How can the REBAHS-LPSP-II program be strengthened to improve access and quality of care at PHCs?
8) What are the key lessons learned from the implementation of the REBAHS-LPSP-II program?

    Probe: how might these lessons be applied to similar programs in the future?
