# Supplementary material for: Mixed method program impact evaluation: Reducing economic barriers to accessing health services (REBAHS) long-term primary healthcare subsidization protocol (LPSP) II action in Lebanon
Source: PLOS Glob Public Health. 2025 Dec 5;5(12):e0005569. doi: 10.1371/journal.pgph.0005569 (PMC12680163; doi:10.1371/journal.pgph.0005569)

S6 Appendix. Segmented Analyses of Mammogram Screening and Full Fasting Lipid Profile. The denominator was used as a contextualizing factor, as the exact figure of eligible persons in the population was not available. ‘All persons’ represents unique individuals (non-repetitive) visiting PHCCs; ‘Persons’ represents unique individuals using the specific service.

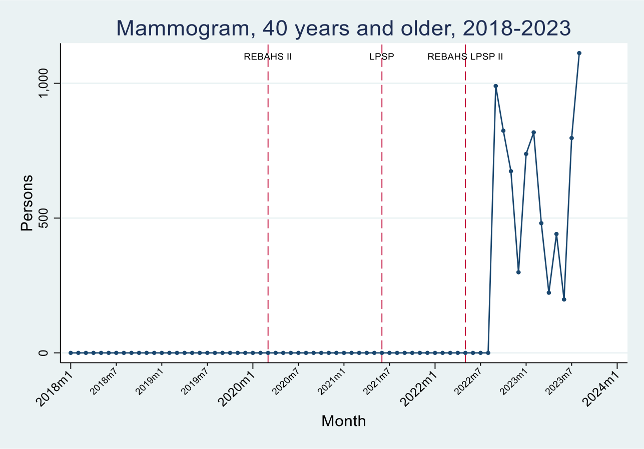

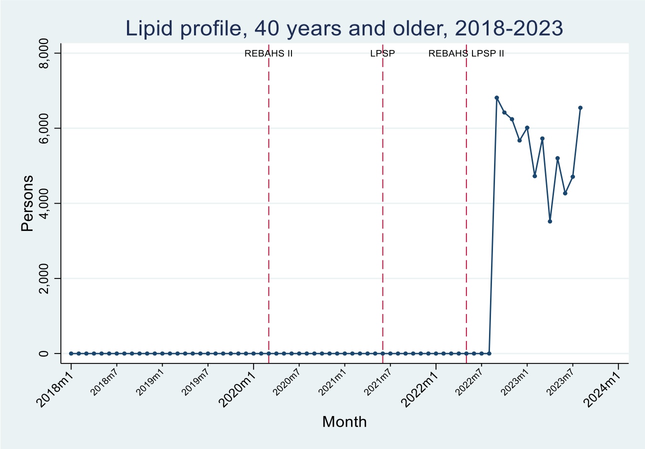

Supplement: S6 Appendix — (PDF) [file pgph.0005569.s006.pdf]
