# Supplementary material for: Mixed method program impact evaluation: Reducing economic barriers to accessing health services (REBAHS) long-term primary healthcare subsidization protocol (LPSP) II action in Lebanon
Source: PLOS Glob Public Health. 2025 Dec 5;5(12):e0005569. doi: 10.1371/journal.pgph.0005569 (PMC12680163; doi:10.1371/journal.pgph.0005569)

S7 Appendix. Interrupted time series analysis for malnutrition screening, pep smear screening, breast feeding counselling, family planning counselling, antenatal care consultations. In the tables presented 'NA' indicates no baseline for comparison, while '-' denotes no significant change.

Malnutrition Screening

	**REBAHS II Impact**	**LPSP Impact**	**REBAHS LPSP II Impact**
**Starting level**	NA	3283	-
**Level change at 1-month post-intervention**	NA	-	-
**Monthly trend, pre-intervention**	NA	-	-
**Monthly trend, post-intervention**	NA	-	-154
**Monthly trend, change**	NA	-	-
**Interpretation**	*NA*	*None*	*None*

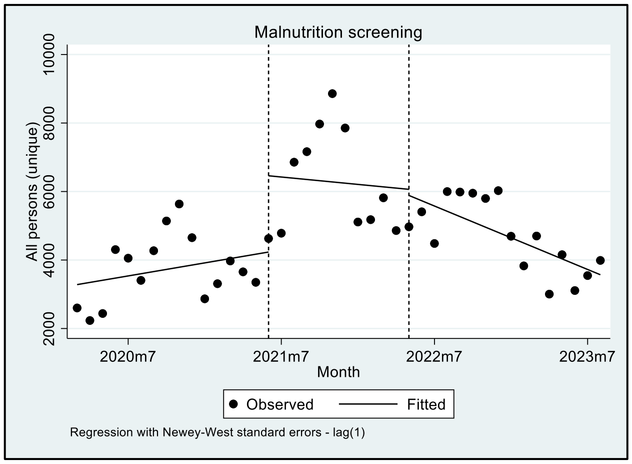

Pap Smear Screening

	**REBAHS II Impact**	**LPSP Impact**	**REBAHS LPSP II Impact**
**Starting level**	NA	120.8	-
**Level at 1-month post-intervention**	NA	353.1	-470.2
**Monthly trend, pre-intervention**	NA	-12.6	-
**Monthly trend, post-intervention**	NA	73.7	-79.7
**Monthly trend, change**	NA	61.1	-
**Interpretation**	***NA***	***Increased level and trend***	***Decreased level***

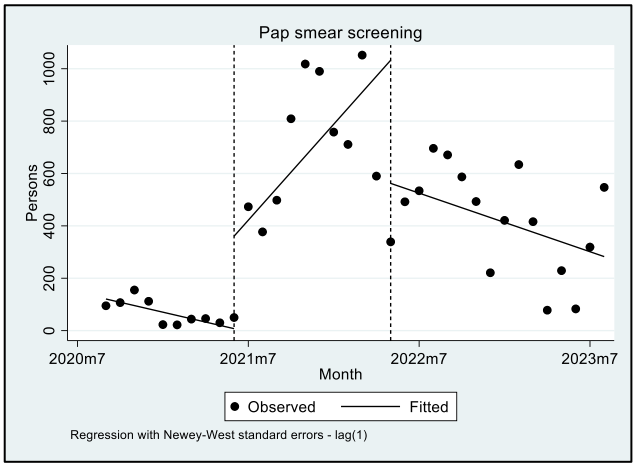

Breastfeeding Counseling

	REBAHS II Impact	LPSP impact	REBAHS LPSP II Impact
**Starting level**	-	-	-
**Level change at 1-month post-intervention**	**-**	**-**	**-**
**Monthly trend, pre-intervention**	-	-	-
**Monthly trend, post-intervention**	9.7	-	-4.4
**Monthly trend, change**	9.2	-11.2	-
**Interpretation**	***Increased trend***	***Decreased trend***	***None***

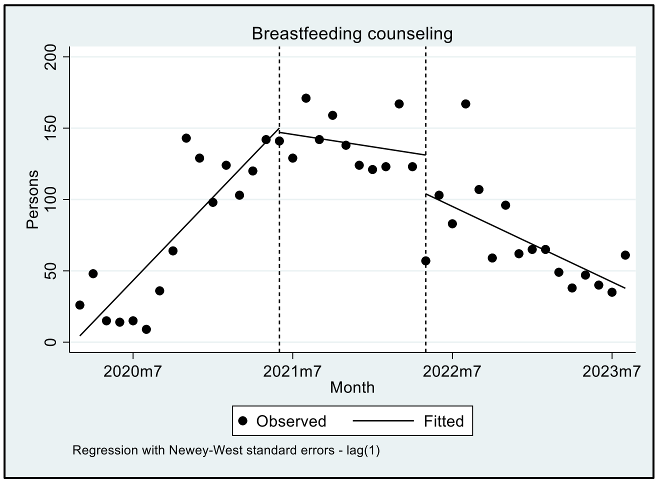

Family Planning Counseling

	REBAHS II Impact	LPSP Impact	REBAHS LPSP II Impact
**Starting level**	*NA*	170.1	-
**Level change at 1-month post-intervention**	*NA*	-	-100.7
**Monthly trend, pre-intervention**	*NA*	-	-
**Monthly trend, post-intervention**	*NA*	-	13.0
**Monthly trend, change**	*NA*	-	11.7
**Interpretation**	***NA***	***None***	***Decreased level & increased trend***

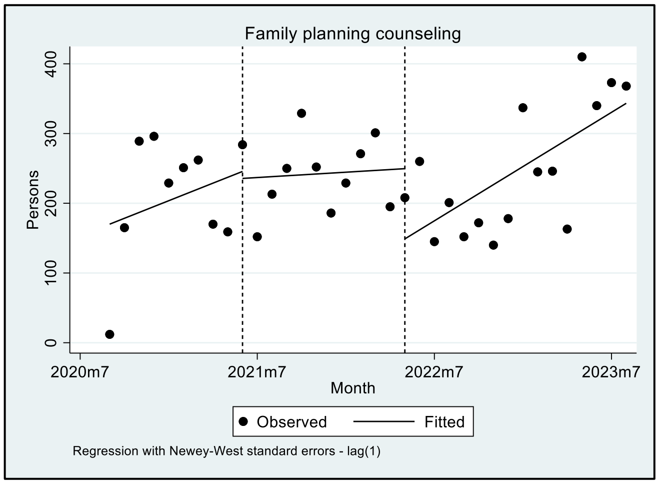

Antenatal Care Consultations

	REBAHS II Impact	LPSP Impact	REBAHS LPSP II Impact
**Starting level**	*NA*	409.4	-
**Level change at 1-month post-intervention**	*NA*	-	-
**Monthly trend, pre-intervention**	*NA*	11.2	-
**Monthly trend, post-intervention**	*NA*	17.5	-
**Monthly trend, change**	*NA*	-	-18.6
**Interpretation**	***NA***	***None***	***Decreased trend***

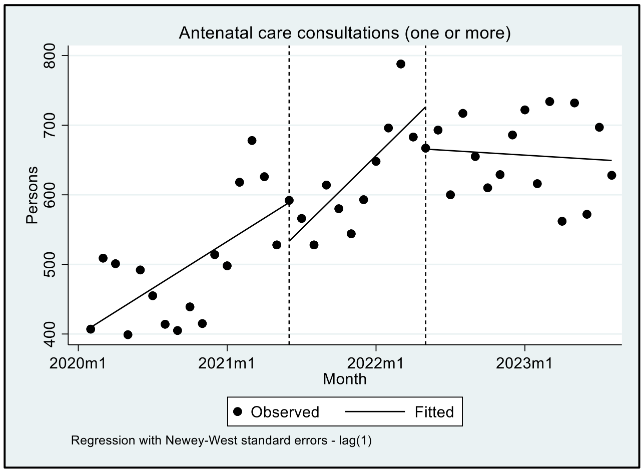

Supplement: S7 Appendix — (PDF) [file pgph.0005569.s007.pdf]
