# Supplementary material for: Mixed method program impact evaluation: Reducing economic barriers to accessing health services (REBAHS) long-term primary healthcare subsidization protocol (LPSP) II action in Lebanon
Source: PLOS Glob Public Health. 2025 Dec 5;5(12):e0005569. doi: 10.1371/journal.pgph.0005569 (PMC12680163; doi:10.1371/journal.pgph.0005569)

S8 Appendix. Interrupted time series analysis for hypertension package, diabetes package, coronary artery disease package, COPD package, nationality Lebanese, ages 0-2 years, proportion, ages 3-9 years, proportion, ages 45-64 years, proportion, ages ≥65 years, proportion.

Hypertension Package

	REBAHS II Impact	LPSP Impact	REBAHS LPSP II Impact
**Starting level**	490.9	-	-
**Level change at 1-month post-intervention**	-	-	-565.0
**Monthly trend, pre-intervention**	-11.1	-	-
**Monthly trend, post-intervention**	70.5	-	-
**Monthly trend, change**	59.3	-	-
**Interpretation**	***Increased trend***	***None***	***Decreased level***

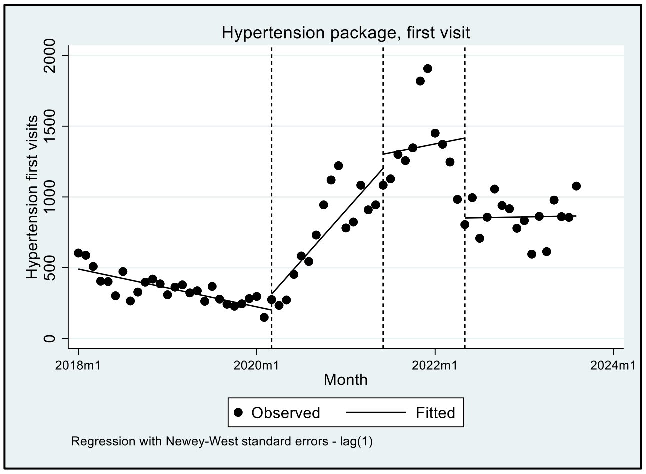

Diabetes Package

	REBAHS II Impact	LPSP Impact	REBAHS LPSP II Impact
**Starting level**	429.5	-	-
**Level change at 1-month post-intervention**	169.3	-	-
**Monthly trend, pre-intervention**	-10.4	-	-
**Monthly trend, post-intervention**	31.6	-	-
**Monthly trend, change**	42.0	-	-
**Interpretation**	***Increased trend & level***	***None***	***None***

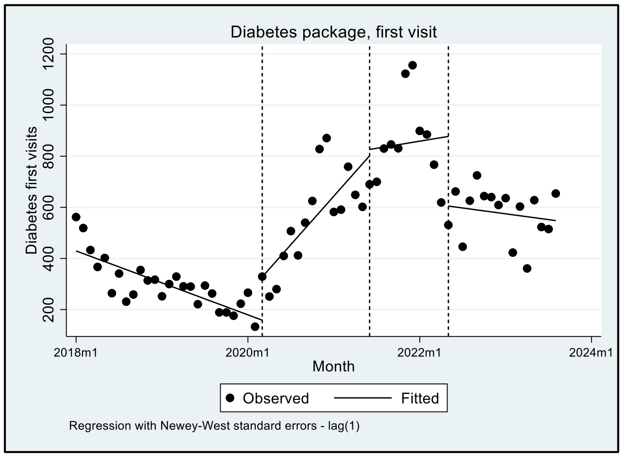

Coronary Artery Disease Package

	REBAHS II Impact	LPSP Impact	REBAHS LPSP II Impact
**Starting level**	21.6	-	-
**Level change at 1-month post-intervention**	-	75.1	-44.9
**Monthly trend, pre-intervention**	-	-	-
**Monthly trend, post-intervention**	2.0	-	-
**Monthly trend, change**	2.3	-	-
Interpretation	***Increased trend***	***Increased level***	***Decreased level***

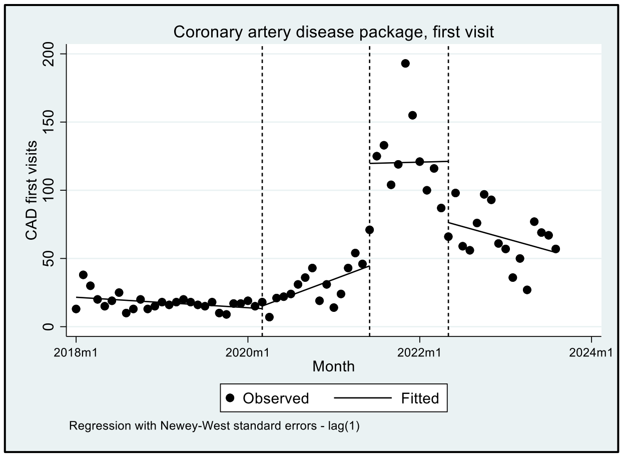

COPD Package

	REBAHS II Impact	LPSP Impact	REBAHS LPSP II Impact
**Starting level**	*NA*	5.0	-
**Level change at 1-month post-intervention**	*NA*	-	-13.0
**Monthly trend, pre-intervention**	*NA*	-	-
**Monthly trend, post-intervention**	*NA*	-	-
**Monthly trend, change**	*NA*	-	-
**Interpretation**	***NA***	***None***	***Decreased level***

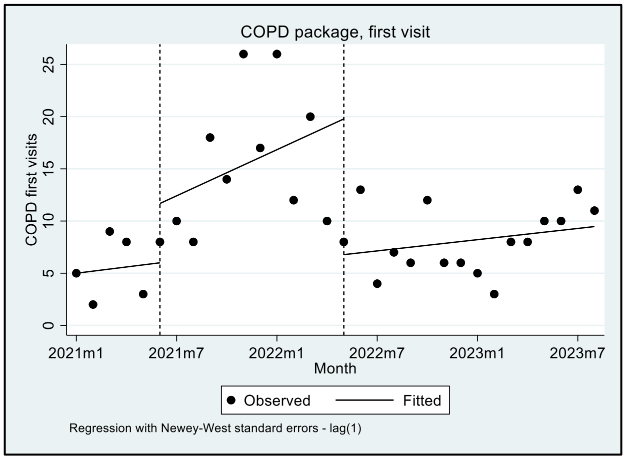

Nationality Lebanese

	REBAHS II Impact	LPSP Impact	REBAHS LPSP II Impact
**Starting level**	25.4	-	-
**Level change at 1-month post-intervention**	-	-	-7.9
**Monthly trend, pre-intervention**	0.7	-	-
**Monthly trend, post-intervention**	0.8	1.3	-0.4
**Monthly trend, change**	0.1	-	-1.7
Interpretation	***Increased trend***	***None***	***Decreased level & trend***

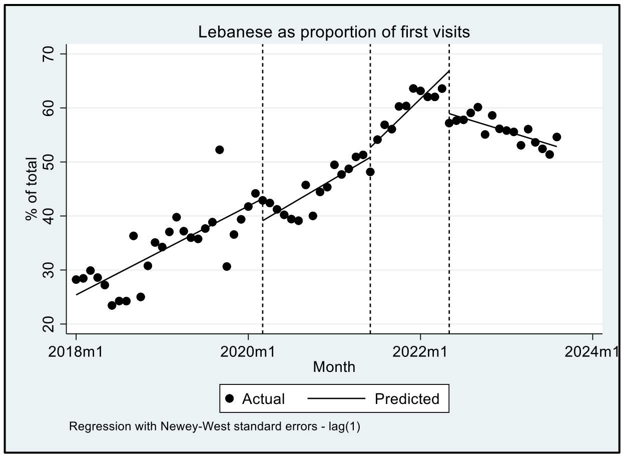

Ages 0-2 years, proportion

	REBAHS II Impact	LPSP Impact	REBAHS LPSP II Impact
**Starting level**	23.88	-	-
**Level change at 1-month post-intervention**	4.26	2.65	2.53
**Monthly trend, pre-intervention**	-	-	-
**Monthly trend, post-intervention**	-0.81	0.36	0.39
**Monthly trend, change**	-0.68	-0.46	0.75
**Interpretation**	***Increased level & decreased trend***	***Increased level & decreased trend***	***Increased level & increased trend***

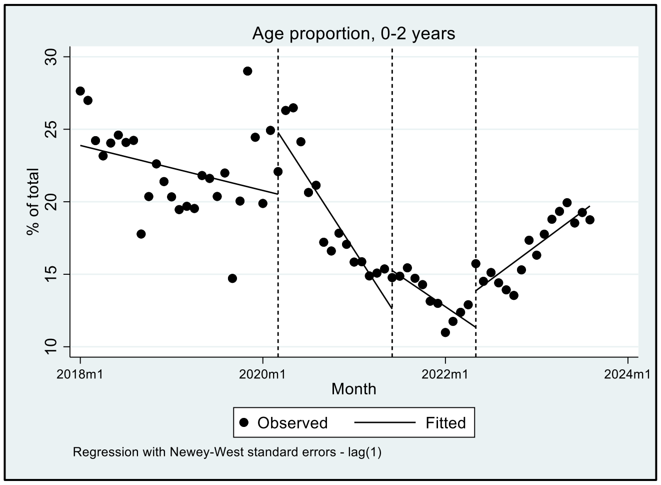

Ages 3-9 years, proportion

	REBAHS II Impact	LPSP Impact	REBAHS LPSP II Impact
**Starting level**	22.69	-	-
**Level change at 1-month post-intervention**	-6.40	-	-
**Monthly trend, pre-intervention**	-	-	-
**Monthly trend, post-intervention**	-	-	-0.3
**Monthly trend, change**	-	-	-
Interpretation	***Decreased level***	***None***	***None***

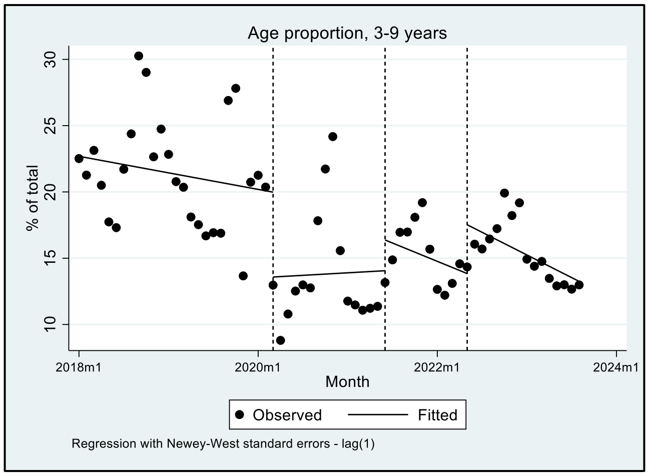

Ages 45-64 years, proportion

	REBAHS II Impact	LPSP Impact	REBAHS LPSP II Impact
**Starting level**	9.71	-	-
**Level change at 1-month post-intervention**	-	-2.84	-3.81
**Monthly trend, pre-intervention**	-	-	-
**Monthly trend, post-intervention**	0.39	0.37	-
**Monthly trend, change**	0.32	-	-0.39
Interpretation	***Increased trend***	***Decreased level***	***Decreased trend & level***

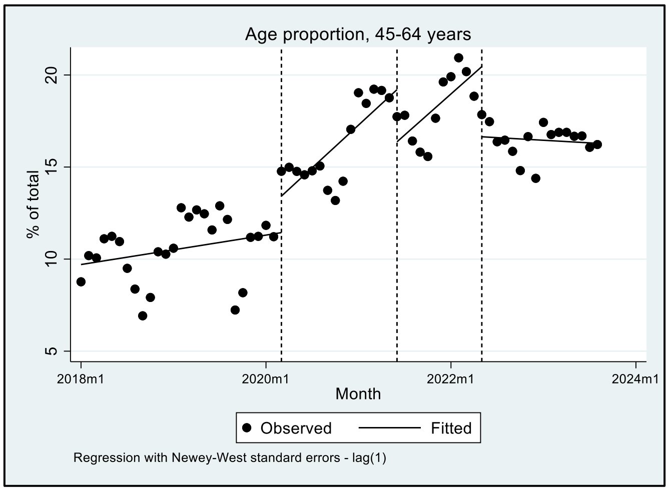

Ages ≥65 years, proportion

	REBAHS II Impact	LPSP Impact	REBAHS LPSP II Impact
**Starting level**	2.97	-	-
**Level change at 1-month post-intervention**	-	-1.69	-3.18
**Monthly trend, pre-intervention**	-	-	-
**Monthly trend, post-intervention**	0.30	0.31	-
**Monthly trend, change**	0.28	-	-0.33
**Interpretation**	***Increased trend***	***Decreased level***	***Decreased trend & level***

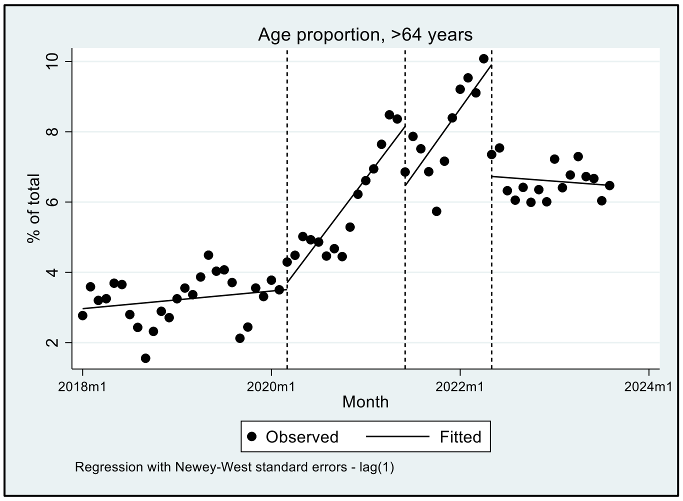

Supplement: S8 Appendix — (PDF) [file pgph.0005569.s008.pdf]
